# Adipose cells promote resistance of breast cancer cells to trastuzumab-mediated antibody-dependent cellular cytotoxicity

**DOI:** 10.1186/s13058-015-0569-0

**Published:** 2015-04-24

**Authors:** Minh Ngoc Duong, Aurore Cleret, Eva-Laure Matera, Kamel Chettab, Doriane Mathé, Sandrine Valsesia-Wittmann, Béatrice Clémenceau, Charles Dumontet

**Affiliations:** Centre de Recherche en Cancérologie de Lyon (CRCL), INSERM UMR 1052, CNRS 5286, 8 Avenue Rockefeller, 69008 Lyon, France; Centre Léon Bérard, Pôle des sciences cliniques, Plateforme d’innovations en immunomonitoring et immunothérapie, 28 Promenade Léa et Napoléon Bullukian, 69008 Lyon, France; INSERM U892, Centre de Recherche en Cancérologie Nantes-Angers, 8 quai Moncousu, BP 70721, 44007 Nantes Cedex, France; Centre Hospitalier Universitaire de Nantes, 1 Place Alexis-Ricordeau, 44000 Nantes, France; Hospices Civils de Lyon, 165 Chemin du Grand Revoyet, 69310 Pierre-Bénite, France

## Abstract

**Introduction:**

Trastuzumab has been used in the treatment of human epidermal growth factor receptor 2 (HER2)-expressing breast cancer, but its efficacy is limited by *de novo* or acquired resistance. Although many mechanisms have been proposed to explain resistance to trastuzumab, little is known concerning the role of the tumor microenvironment. Given the importance of antibody-dependent cellular cytotoxicity (ADCC) in the antitumor effect of trastuzumab and the abundance of adipose tissue in the breast, we investigated the impact of adipocytes on ADCC.

**Methods:**

We set up a coculture system to study the effect of adipocytes on ADCC *in vitro*. The results were validated *in vivo* in a mouse xenograft model.

**Results:**

We found that adipocytes, as well as preadipocytes, inhibited trastuzumab-mediated ADCC in HER2-expressing breast cancer cells via the secretion of soluble factors. The inhibition of ADCC was not due to titration or degradation of the antibody. We found that adipose cells decreased the secretion of interferon-γ by natural killer cells, but did not alter natural killer cells’ cytotoxicity. Preincubation of breast cancer cells with the conditioned medium derived from adipocytes reduced the sensitivity of cancer cells to ADCC. Using a transcriptomic approach, we found that cancer cells undergo major modifications when exposed to adipocyte-conditioned medium. Importantly, breast tumors grafted next to lipomas displayed resistance to trastuzumab in mouse xenograft models.

**Conclusions:**

Collectively, our findings underline the importance of adipose tissue in the resistance to trastuzumab and suggest that approaches targeting the adipocyte–cancer cell crosstalk may help sensitize cancer cells to trastuzumab-based therapy.

**Electronic supplementary material:**

The online version of this article (doi:10.1186/s13058-015-0569-0) contains supplementary material, which is available to authorized users.

## Introduction

Human epidermal growth factor receptor 2 (HER2) is amplified in 15% to 20% of breast cancers, and its overexpression is associated with adverse prognosis [1]. Trastuzumab, a humanized monoclonal antibody directed against HER2, was approved in 1998 for the treatment of HER2-overexpressing breast cancer. Mechanisms of action of trastuzumab include inhibition of HER2 dimerization, direct induction of cell growth arrest and apoptotic cell death, inhibition of HER2 shedding, and recruitment of immune effector cells to mediate tumor lysis [2]. This latter mechanism, designated as antibody-dependent cellular cytotoxicity (ADCC), has been shown to be dependent on expression of Fc receptors (FcRs) by innate immune cells [3]. Recent studies by Park *et al.* also demonstrated the involvement of adaptive immune cells in the action of anti-HER2/neu antibody [4]. As a single agent or in combination with chemotherapy, trastuzumab has shown remarkable efficacy [5]. However, not all patients respond to trastuzumab and some patients whose breast cancer initially responds to treatment eventually experience progression, corresponding to primary and acquired resistance to trastuzumab, respectively [5].

Different mechanisms of resistance to trastuzumab have been reported. Downregulation of phosphatase and tensin homolog (PTEN), a phosphatase whose activation contributes to trastuzumab activity, has been shown to confer trastuzumab resistance both *in vitro* and *in vivo* [6]. Patients with PTEN deficiency displayed poorer responses to trastuzumab-based therapy than those with active PTEN [6]. Shedding of the extracellular domain of HER2 protein by proteolytic cleavage has been shown to neutralize the antitumor effects of trastuzumab [7]. Elevated circulating levels of HER2 have also been correlated with disease progression in patients treated with trastuzumab-based therapy [8]. Furthermore, masking of the HER2 antigens by the glycoprotein mucin 4 (MUC4) has been shown to reduce binding of anti-HER2 antibodies *in vitro* [9], whereas increased MUC4 levels have been observed *in vivo* in tumors that were resistant to anti-HER2 therapies [10]. Additionally, activation of other signaling pathways, notably insulin-like growth factor 1 (IGF-1) receptor, has also been reported to inhibit trastuzumab-mediated growth inhibition in breast cancer cells [11].

The aforementioned mechanisms of resistance are related to alterations in the tumor cells themselves and do not take into account the impact of the tumor microenvironment. This latter phenomenon is highly complex in terms of cellular composition with different cell types, including adipocytes, preadipocytes, endothelial cells, pericytes and immune cells. Several studies have shown that immune suppressor cells, such as tumor-associated macrophages, myeloid-derived suppressor cells and regulatory T cells, are recruited to the tumor sites and promote immune evasion [12-14]. However, the implication of resident cells, notably adipocytes, in tumor resistance to trastuzumab remains largely unknown.

Adipocytes are the most abundant cells in the breast adipose tissue. It has been shown that adipocytes are not simply energy storage depots but also active sources of various paracrine and endocrine factors, termed *adipocytokines*, such as leptin, adiponectin, tumor necrosis factor (TNF)-α and interleukin (IL)-6 [15]. Given their abundance and their proximity to tumor cells, adipocytes have been shown to affect tumor behavior, including tumor growth and metastasis [16]. Moreover, recent studies suggested the implication of adipocytes in resistance to radiotherapy [17] and chemotherapy [18]. Furthermore, obesity, which is associated with increased fat mass and inflammatory adipose tissue phenotype, has been shown to be an important risk factor for breast cancer in postmenopausal women [19]. Chemotherapy and endocrine therapy have also been observed to be less effective in patients with breast cancer and obesity [20].

Adipocytes derive from precursors, termed *preadipocytes*, through a tightly controlled differentiation process. These preadipocytes are known to possess a secretion profile distinct from that of adipocytes [21]. Increased evidence has shown that preadipocytes contribute to tumor progression and resistance to chemotherapy [22].

In this study, we evaluated whether preadipocytes and adipocytes could contribute to resistance to trastuzumab. We show that preadipocytes and adipocytes inhibited trastuzumab-mediated ADCC in breast cancer cells via secretion of soluble factors, resulting in a direct protective effect on tumor cells.

## Methods

### Cell culture

BT-474, SK-BR-3, MDA-MB-453, and MDA-MB-361 human breast cancer cell lines, all obtained from the American Type Culture Collection (Manassas, VA, USA), were cultured in complete RPMI 1640 medium (supplemented with 10% fetal calf serum (FCS), 2 mM l-glutamine, 100 U/ml penicillin and 100 μg/ml streptomycin). Human multipotent adipose-derived stem cells (hMADS) were provided by Dr Christian Dani, UMR 6543 CNRS, Nice, France, with approval granted by the local ethics committee of the University of Nice. hMADS were cultured in complete Dulbecco’s modified Eagle’s medium (DMEM) supplemented with 2.5 ng/ml basic fibroblast growth factor (FGF2). The immortalized human mammary epithelial hTERT-HME1 cells were cultured in complete DMEM/F-12 medium supplemented with 1% nonessential amino acids, 50 μg/ml gentamicin, 3.5 μg/ml insulin, 100 ng/ml epidermal growth factor and 500 ng/ml hydrocortisone. All cells were maintained at 37°C in presence of 5% CO_2_, except in hypoxic experiments, in which cells were maintained in 1% O_2_.

### Induction of human multipotent adipose-derived stem cell differentiation

The differentiation of hMADS cells was previously described [23,24]. Differentiated hMADS (#hMADS) were used at day 14 of differentiation. The differentiation yield was estimated to range between 70% and 80% and was verified by Oil Red O staining, as described previously [25]. hMADS and #hMADS were incubated in culture media containing 10% FCS for 2 days, and the conditioned media (CM) were harvested after centrifugation at 300 × *g* for 5 minutes and frozen at −20°C before use.

### Retroviral transduction of NK-92 natural killer cells

NK-92, the human natural killer (NK) cell line [26], generously provided by Conkwest (Del Mar, CA, USA), was grown in complete RPMI 1640 culture medium. NK-92-CD16 cells were obtained by transduction of pMX/CD16 plasmid [27], using retroviral supernatant as described in Additional file 1.

### Isolation and differentiation of human adipose-derived stem cells

Adipose tissues were provided by Dr Emmanuel Delay (Centre Léon Bérard, Lyon, France). They were obtained by liposuction from abdominal fat of patients undergoing plastic surgery. Written patient consent was obtained, and this protocol was approved by the Lyon research ethics committee. Adipose tissue samples (5 to 10 g) were rapidly digested with 100 collagen digestion units/ml collagenase (Sigma-Aldrich, St Louis, MO, USA) at 37°C with agitation for 30 minutes. Digestion was stopped by addition of complete DMEM/F-12 medium. After centrifugation at 300 × *g* for 7 minutes, cells corresponding to the stromal vascular fraction were seeded for adherence and amplification in complete DMEM/F-12 medium supplemented with 10 ng/ml FGF2. Adipose-derived stem cells (ASCs) were used at passage 2 or 3 and verified by flow cytometry to be CD14−, CD45−, CD73+, CD90+, CD105+ and HLA-ABC+ (human leukocyte antigen abacavir). Differentiation of ASCs into adipocytes was performed as described previously [28].

### Antibody-dependent cellular cytotoxicity assay

Human mammary epithelial hTERT-HME1 and hMADS cells were seeded at 1.2 × 10^5^ cells/ml/well in 12-well plates. The medium of 12-day #hMADS was changed and supplemented with 10% FCS. Two days later, target cells were labeled with 12.5 μM calcein-AM (Sigma-Aldrich) for 30 minutes at 37°C and added at 10^5^ cells/0.5 ml/well. NK-92-CD16 cells were added at 5 × 10^5^ cells/0.5 ml/well (effector to target (ratio = 5:1), with trastuzumab at 1 μg/ml final concentration. After 4 hours of incubation at 37°C, the supernatants in each well were harvested and measured for fluorescence signals at 485/535 nm. Triton X-100 was added to the control wells to estimate total cell lysis. Cytotoxicity was calculated using the following formula: percent cytotoxicity = (experimental lysis − spontaneous lysis) ÷ (maximal lysis − spontaneous lysis) × 100.

### Flow cytometry

Cells were labeled with corresponding antibodies for 15 minutes at room temperature and analyzed with a BD LSR II flow cytometer using BD FACSDiva software (BD Biosciences, San Diego, CA, USA) and FlowJo software (Tree Star, Ashland, OR, USA). The antibodies used are detailed in Additional file 1.

### Enzyme-linked immunosorbent assay

Supernatants obtained from NK cells cultured alone or from ADCC assays in the presence of adipose cells were subjected to enzyme-linked immunosorbent assay (R&D Systems, Minneapolis, MN, USA) according to the manufacturer’s instructions.

### Fluorescence microscopy

BT-474 cells were seeded overnight onto Nunc Lab-Tek chambered coverglasses (Thermo Fisher Scientific, USA). CM from #hMADS or hMADS cells were added, and cells were incubated for additional 4 hours at 37°C before being labeled on ice with anti-ErbB 2 Affibody fluorescein isothiocyanate (FITC) (Abcam, Cambridge, UK) for 15 minutes. After the washing, cells were observed with a confocal Zeiss LSM 780 microscope (Carl Zeiss, Oberkochen, Germany).

### Microarray

BT-474 cells or SK-BR-3 cells were exposed to #hMADS-CM for 2 hours. RNA was extracted using the QIAamp RNeasy Mini Kit (QIAGEN, Valencia, CA, USA). After RNA amplification with the Illumina TotalPrep RNA Amplification Kit (Life Technologies, Carlsbad, CA, USA), cRNA was hybridized on HumanHT-12 v4 Expression BeadChips (Illumina, San Diego, CA, USA). Scanning was performed with an Illumina iScan microarray scanner, and data were analyzed with GeneSpring and Ingenuity software (Agilent Technologies, Santa Clara, CA, USA). Our microarray data have been deposited in the Gene Expression Omnibus database under accession number [GEO:GSE52660].

### Reverse transcription and quantitative PCR

BT-474 cells were exposed to #hMADS-CM or hMADS-CM for the Additional file 2: Figure S6. Cells were harvested, and RNA was extracted using the RNeasy Mini Kit (QIAGEN). Reverse transcription (RT) was performed using random primers (Life Technologies). Quantitative PCR (qPCR) was performed with primers (QIAGEN) of the Additional file 2: Figure S6 using the LightCycler Nano Instrument (Roche Life Science, Indianapolis, IN, USA).

### Western blot analysis

BT-474 cells were exposed to either #hMADS-CM or control medium for the Figure 5D. Proteins were extracted in radioimmunoprecipitation assay buffer and subjected to SDS-PAGE and immunoblot analysis. The antibodies used were anti-phospho-Akt and anti-Akt (Cell Signaling Technology, Beverly, MA, USA) and anti-tubulin (Sigma-Aldrich).

### *In vivo* studies

All animal procedures were performed in accordance with European Union directive 86/609/EEC. Experiments were performed under individual permit and in animal care facilities accredited by the French Ministry of Agriculture. The study was approved by the local animal ethics committee (Université Claude Bernard Lyon I, protocol number BH-2012-40). The study was conducted using severe combined immunodeficiency (SCID) mice, with four used per group. Each mouse was given a subcutaneous injection of 1 ml of abdominal adipose tissue obtained from patients undergoing plastic surgery to form a lipoma. After 1 week, BT-474 tumors were grafted subcutaneously in contact with the lipoma. Treatments were initiated when the tumor volume was 100 mm^3^, with intraperitoneal administration of antibodies (rituximab 30 mg/kg or trastuzumab 25 mg/kg, twice per week for 3 weeks). Tumor growth was directly measured using a caliper, based on the difference of consistencies between the lipoma and the tumor.

### Statistical analysis

All experiments were performed at least three times. Mean ± SD values of representative experiments are shown. Statistical significance was evaluated using paired Student’s *t*-tests on the means of at least three independent *in vitro* experiments. Unpaired Student’s *t*-tests were used for *in vivo* experiments. *P*-values <0.05 were deemed significant.

## Results

### hMADS and differentiated hMADS inhibit trastuzumab-mediated antibody-dependent cellular cytotoxicity

**Figure 1:**
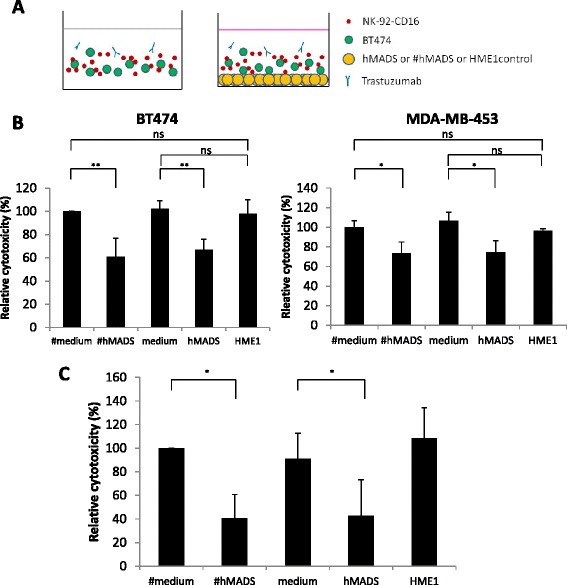
#hMADS adipocytes and hMADS preadipocytes inhibit antibody-dependent cellular cytotoxicity. **(A)** Schema of antibody-dependent cellular cytotoxicity (ADCC) assays in the absence (left) or the presence (right) of adipose cells. **(B)** ADCC assays on BT-474 and MDA-MB-453 cells as described in **(A)**. **(C)** ADCC assay on BT-474 cells performed in hypoxic conditions with 1% O_2_. The cytotoxicity was normalized to the control differentiated medium as 100%. Mean ± SD values of three independent experiments, each performed in triplicate, are shown **(B** and **C)**. hMADS, Human multipotent adipose-derived stem cells; #hMADS, Differentiated human multipotent adipose-derived stem cells; HME, Human mammary epithelial cell. *P < 0.05; **P < 0.01; ns, Not significant.

To investigate the role of preadipocytes and adipocytes, we performed ADCC assays on HER2-positive (HER2+) estrogen receptor (ER)-positive human breast cancer cells (BT-474) and NK cells (NK-92-CD16) in the presence of undifferentiated hMADS or #hMADS cells (corresponding to models of preadipocytes and adipocytes, respectively) or their control media (Figure 1A). NK-92-CD16 cells alone did not exhibit any cytotoxicity on BT-474 cells in the absence of trastuzumab during the 4-hour assays (Additional file 3: Figure S1A). As shown in Figure 1B and Additional file 3: Figure S1A, both hMADS and #hMADS reduced trastuzumab-mediated ADCC by approximately 30% as compared with the control media. In contrast, no inhibition of ADCC was observed in the presence of immortalized human mammary epithelial hTERT-HME1 cells (Figure 1B), indicating that the inhibition was not due to the physical presence of a feeder cell layer, but was specific to adipose cells. Similar results on the adipose-mediated inhibition of ADCC were obtained with the HER2+/ER− MDA-MB-453 cells (Figure 1B), suggesting that the inhibitory effect of adipose cells on ADCC was independent of the ER status on cancer cells. We also found that adipose cells inhibited ADCC to a lesser extent with HER2+/ER− SK-BR-3 cells, but not with HER2+/ER+ MDA-MB-361 cells (Additional file 3: Figure S1B). This was not related to the difference in levels of HER2 expression between these cell lines (Additional file 4: Figure S2). Therefore, we chose BT-474 cells as target cells for later experiments. Collectively, these results indicate that adipose cells (both hMADS and #hMADS) inhibited trastuzumab-mediated ADCC.

Because hypoxia is an important feature that characterizes the tumor sites [29], we also performed our experiments under hypoxic conditions with 1% O_2_. Interestingly, under hypoxic conditions, hMADS and #hMADS inhibited ADCC even more markedly (up to 60%) than under normoxic conditions (Figure 1C). This result highlights the relevance of adipose cells in the tumor microenvironment for the protection of cancer cells from trastuzumab-mediated lysis of tumor cells by NK cells.

### Inhibition of antibody-dependent cellular cytotoxicity is mediated by soluble factors

**Figure 2:**
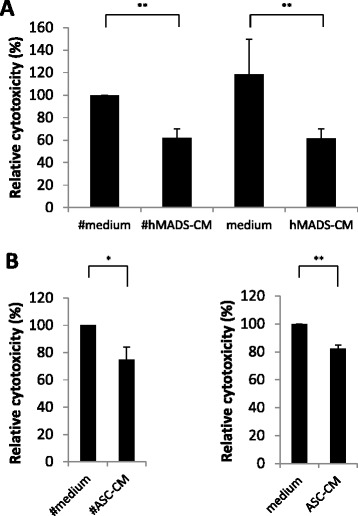
Adipocyte- and preadipocyte-derived soluble factors inhibit antibody-dependent cellular cytotoxicity. **(A)** Antibody-dependent cellular cytotoxicity (ADCC) assays on BT-474 cells performed in the presence of the conditioned media from differentiated human multipotent adipose-derived stem cells (#hMADS-CM) or human multipotent adipose-derived stem cells (hMADS-CM) cells (vol/vol = 1/1). **(B)** Similar to **(A)**, but the CM were from adipose-derived stem cells (ASC-CM) or *in vitro* differentiated #ASCs (#ASC-CM) obtained from abdominal adipose tissue. Data represent the means ± SD of three independent experiments, each performed in triplicate. *P < 0.05; **P < 0.01.

To investigate whether the inhibition of ADCC by hMADS and #hMADS was dependent on cell contact or mediated by soluble factors, we used CM from hMADS and #hMADS (hMADS-CM and #hMADS-CM, respectively) in our ADCC assays. As shown in Figure 2A, both hMADS-CM and #hMADS-CM inhibited ADCC at levels similar to those observed in cell coculture experiments. Moreover, when the CM from primary human ASCs or from *in vitro* differentiated ASCs (#ASCs) were used, we also obtained an inhibition of ADCC (Figure 2B). These data indicate that both preadipocytes and adipocytes secreted soluble factors that inhibited ADCC.

In order to determine the nature of the inhibition, we performed a kinetic study of ADCC. The inhibition of ADCC by hMADS-CM and #hMADS-CM was observed at early times (1 hour after the coincubation) (Additional file 5: Figure S3A). Interestingly, treatment of #hMADS-CM with proteinase K reversed the inhibition of ADCC (Additional file 5: Figure S3B), suggesting that the inhibitory factors are likely proteins.

### Inhibition of antibody-dependent cellular cytotoxicity is not caused by a sequestration or degradation of the antibody

**Figure 3:**
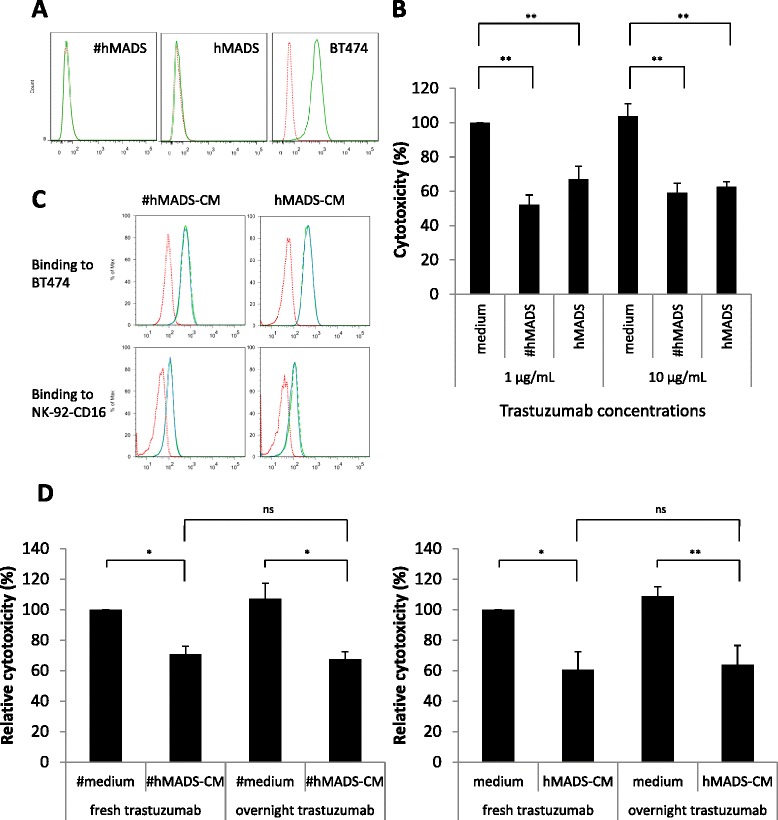
#hMADS adipocytes and hMADS preadipocytes do not alter trastuzumab. **(A)** Binding of fluorescein isothiocyanate (FITC)-conjugated trastuzumab to BT-474, differentiated human multipotent adipose-derived stem cells (#hMADS) and human multipotent adipose-derived stem cells (hMADS). Dotted red lines indicate unstained cells, and solid green lines indicate cells labeled with FITC-conjugated trastuzumab. **(B)** Antibody-dependent cellular cytotoxicity (ADCC) assays performed with 1 μg/ml or 10 μg/ml trastuzumab in the presence of #hMADS or hMADS. **(C)** Binding of FITC-conjugated trastuzumab, previously incubated in #hMADS-CM or hMADS-CM or in the control media, on BT-474 cells (1 μg/ml) or NK-92-CD16 cells (10 μg/ml). Dotted red lines indicate unstained cells, dashed green lines indicate FITC-conjugated trastuzumab preincubated in the control media and solid blue lines indicate FITC-conjugated trastuzumab preincubated in conditioned media (CM) from hMADS or hMADS (#hMADS-CM or hMADS-CM, respectively). **(D)** ADCC assays performed with fresh trastuzumab or trastuzumab preincubated overnight in the control media or in #hMADS-CM (left) or hMADS-CM (right). Results representative of three independent experiments are shown in **(A)** and **(C)**. Mean ± SD values of three independent experiments, each performed in triplicate, are shown in **(B)** and **(D)**. *P < 0.05; **P < 0.01.

To determine whether preadipocytes and adipocytes inhibit ADCC by sequestering the antibody, we first determined the ability of trastuzumab to bind to these cells. As shown in Figure 3A, whereas trastuzumab bound efficiently to HER2-expressing BT-474 cells, no binding was observed on hMADS or #hMADS. In addition, hMADS and #hMADS showed no or very low expression of the Fcγ receptors CD16, CD32 and CD64 (Additional file 6: Figure S4). Furthermore, a tenfold increase in the concentration of trastuzumab did not abolish the inhibition of ADCC by hMADS or #hMADS (Figure 3B). Together, these results suggest that the inhibition of ADCC by hMADS and #hMADS was not due to sequestration of the antibody.

Because adipose tissue has been shown to express and secrete metalloproteinases [30,31], enzymatic cleavage of trastuzumab is a possible mechanism of neutralization of the antitumor action of this antibody. For this reason, we investigated the possible alteration of trastuzumab by CM from hMADS and #hMADS cells. As shown in Figure 3C, FITC-conjugated trastuzumab that was previously preincubated with hMADS-CM or #hMADS-CM for 4 hours bound to HER2-expressing BT-474 cells and FcR-expressing NK-92-CD16 cells, similarly to non-preincubated antibody. Moreover, trastuzumab preincubated overnight in either hMADS-CM or #hMADS-CM displayed ADCC activity as efficiently as unconditioned antibody (Figure 3D). These results show that there is no degradation of trastuzumab, at least during the time of our studies.

### Natural killer cell cytotoxicity is not altered by preadipocyte- or adipocyte-conditioned media

**Figure 4:**
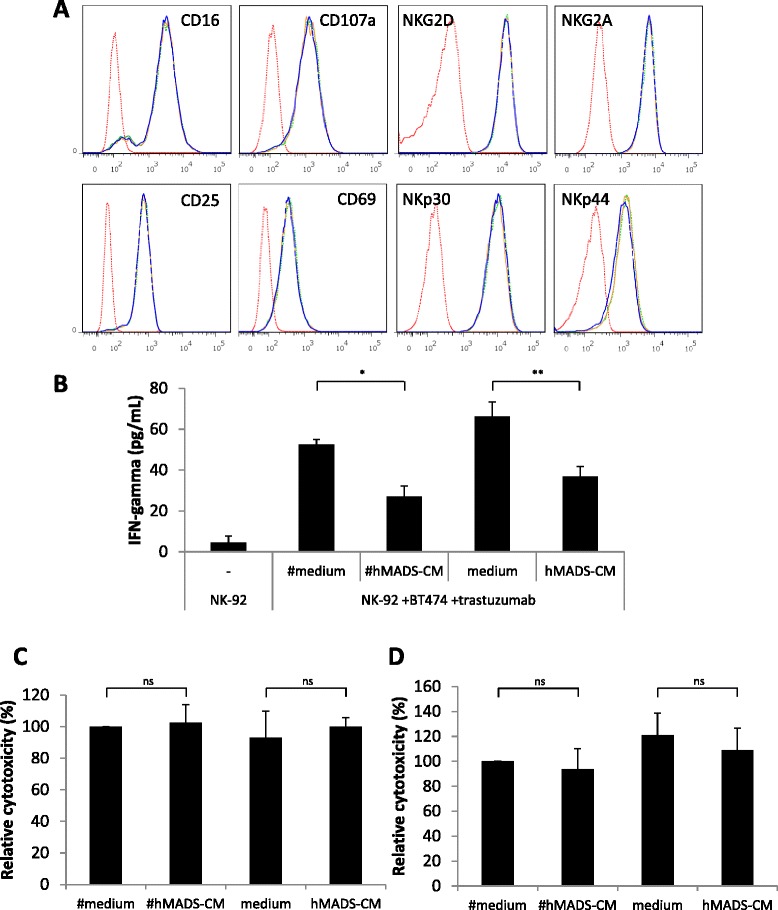
#hMADS-CM and hMADS-CM do not alter natural killer cell cytotoxicity. **(A)** Expression of markers and receptors on NK-92-CD16 cells from antibody-dependent cellular cytotoxicity (ADCC) assays in the presence of conditioned media of differentiated human multipotent adipose-derived stem cells (#hMADS-CM) or human multipotent adipose-derived stem cells (hMADS-CM) or in the control medium. Dotted red lines indicate unstained NK-92-CD16 cells, dotted green lines indicate control medium, solid coral lines indicate #hMADS-CM and dashed blue line indicate hMADS-CM conditions, respectively. **(B)** Enzyme-linked immunosorbent assay of the supernatants from NK-92-CD16 cells after ADCC assay. IFN, Interferon. **(C)** ADCC assays using NK-92-CD16 cells preincubated overnight with #hMADS-CM or hMADS-CM or the control media. **(D)** Cytotoxicity assay using K-562 cells as target cells at an effector to target ratio of 5:1 in the presence of #hMADS-CM, hMADS-CM or their control media. Results representative of three independent experiments are shown in **(A)**. Mean ± SD values of three independent experiments, each performed in duplicate **(B)** or triplicate **(C** and **D)**, are shown. *P < 0.05; **P < 0.01; ns, Not significant.

To investigate whether hMADS- and #hMADS-secreted factors alter NK cell phenotype or functions, we analyzed NK cell markers at the end of the 4-hour ADCC assay. As shown in Figure 4A, no modification of CD16 or CD107a expression levels was observed in the presence of hMADS-CM or #hMADS-CM. Additionally, the expression levels of the activation markers CD25 and CD69—as well as of the other NK receptors, NKG2D, NKp30, NKp44 and NKG2A—were unchanged (Figure 4A). Interestingly, the presence of adipocyte-conditioned media decreased the secretion of interferon (IFN)-γ by NK cells as compared with the control media (Figure 4B). However, when NK-92-CD16 cells were preincubated overnight with hMADS-CM or #hMADS-CM, we did not observe any modification of NK cell viability or of NK cells’ ability to exert their cytotoxicity in trastuzumab-mediated ADCC (Figure 4C and Additional file 7: Figure S5). Furthermore, the adipocyte-conditioned media did not inhibit the spontaneous lysis of K-562 cells by NK-92 cells (Figure 4D). Overall, these data suggest that NK-92-CD16 cells may be altered by hMADS-CM or #hMADS-CM for their secretion of cytokines, but not for their cytotoxicity.

### Conditioned medium from adipocytes reduces sensitivity of tumor cells to antibody-dependent cellular cytotoxicity

**Figure 5:**
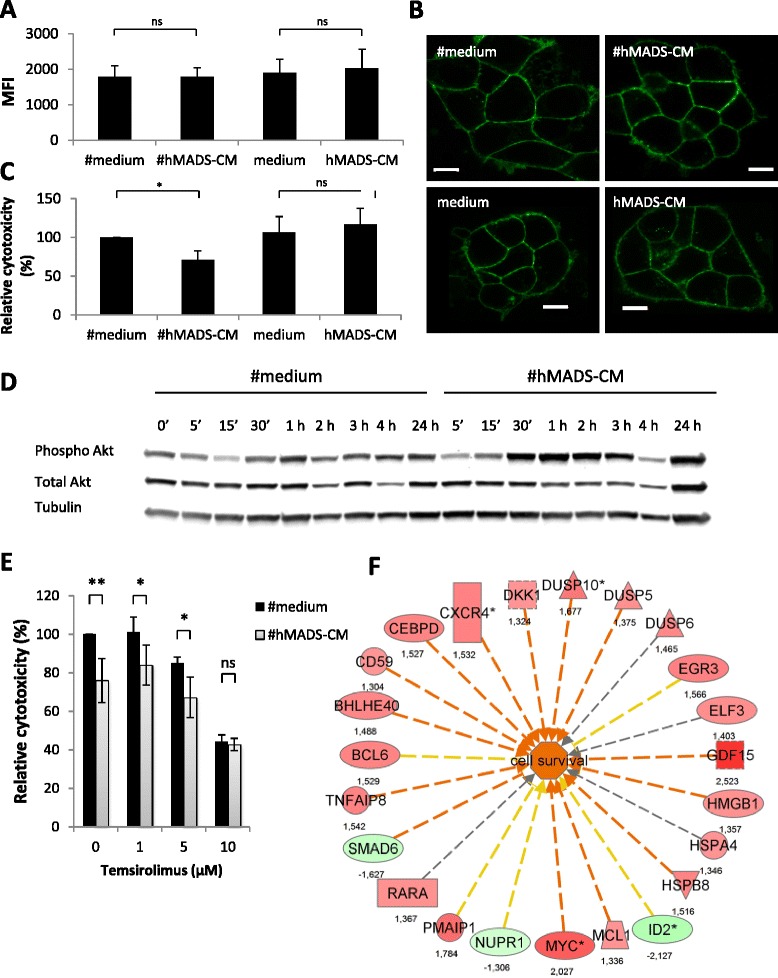
Conditioned media of differentiated human multipotent adipose-derived stem cells increases the resistance of BT-474 cells against ADCC. Human epidermal growth factor receptor 2 (HER2) expression **(A)** or localization **(B)** on BT-474 cells after 4-hour incubation with conditioned media of differentiated human multipotent adipose-derived stem cells (#hMADS-CM) or undifferentiated human multipotent adipose-derived stem cells (hMADS-CM) or their control media. Scale bars indicate 10 μm. **(C)** Antibody-dependent cellular cytotoxicity (ADCC) assays of BT-474 cells preincubated overnight with #hMADS-CM, hMADS-CM or their control media. **(D)** Kinetic induction of Akt phosphorylation (Phospho Akt) in BT-474 cells exposed to #hMADS-CM or the control medium (#medium) for the indicated times. Quantification of the intensity of the bands is shown. Mean ± SD values of three independent experiments are shown in **(A)** and **(C)**. Results representative of three independent experiments are shown in **(B)** and **(D)**. **(E**) Reversion of #hMADS-induced inhibition of ADCC by temsirolimus. Temsirolimus was added at the beginning of ADCC assays at the indicated concentrations. **(F)** Genes involved in cell survival in BT-474 cells after exposure to #hMADS-CM. Numbers correspond to fold changes. Vertical rectangle, G protein–coupled receptor; dashed square, growth factor; inverted triangle, kinase; horizontal rectangle, ligand-dependent nuclear receptor; triangle, phosphatase; oval, transcription regulator; trapezoid, transporter; circle, other. Genes in red or green correspond to upregulated or downregulated, respectively. Red lines predict activation; yellow lines indicate inconsistent downstream effect; and gray lines correspond to unpredicted effect. *P < 0.05; **P < 0.01; ns, Not significant.

We studied whether hMADS-CM and #hMADS-CM had a direct effect on tumor cells to reduce their sensitivity to ADCC. Because an alteration of HER2 on target cancer cells may lead to a decrease in the antitumor effects mediated by trastuzumab, we first evaluated the modification of HER2 expression on BT-474 cells exposed to the adipocyte-conditioned media. As shown in Figure 5A and 5B, no modifications of HER2 expression levels (Figure 5A) or changes in HER2 distribution on plasma membrane (Figure 5B) were observed. Importantly, when BT-474 cells were preincubated overnight with #hMADS-CM, but not with hMADS-CM, BT-474 cells displayed a significant decrease in lysis (Figure 5C), suggesting that the CM from #hMADS cells had a direct effect on cancer cells to decrease their sensitivity to trastuzumab-mediated ADCC. This was supported by our finding that upon exposure to #hMADS-CM, there was rapid phosphorylation (from 30 minutes) of Akt in BT-474 cells (Figure 5D). Interestingly, when temsirolimus, a specific inhibitor of mammalian target of rapamycin (mTOR), which is a main downstream target of activated Akt, was added in ADCC assays in the presence of #hMADS-CM, a reversion of the inhibition of ADCC could be observed at high concentrations of temsirolimus (Figure 5E). Together, these results suggest the implication of the Akt/mTOR pathway in the increase in resistance of BT-474 cells induced by #hMADS-CM.

### Adipocyte-conditioned medium upregulates the expression of survival genes in breast cancer cells

To gain insight into the changes induced in tumor cells by adipocyte-secreted factors, we used a transcriptomic approach on BT-474 cells and SK-BR-3 exposed to #hMADS-CM for 2 hours at 37°C. In BT-474 cells, we found that #hMADS-CM rapidly induced modification of the expression of several genes among more than 47,000 transcripts covered by the microarray (Additional file 8: Table S1), with many of these genes being involved in cell survival (Figure 5F). Among these genes, some were found to be upregulated in both BT-474 and SK-BR-3 cells (*DUSP5*, *MCL1* and *CEBPD*), whereas others were downregulated in both cell lines (*ID2* and *SMAD6*) (Additional file 9: Table S2). Interestingly, *GDF15*, *MYC*, *CXCR4* and *DKK1* were upregulated in BT-474 cells, but not in SK-BR-3 cells, which were previously shown to be less sensitive to #hMADS-induced inhibition of ADCC. We further confirmed by RT-qPCR the kinetic induction of *GDF15*, *MYC* and two alternative splicing variants of *CXCR4* in BT-474 cells upon exposure to #hMADS-CM (Additional file 2: Figure S6). Whereas the expression levels of both *CXCR4* variants rapidly increased and reached their maxima at 1 hour, *GDF15* and *MYC* were induced at maximal levels between 2 and 3 hours after exposure. Intriguingly, separate downregulation of *GDF15*, *MYC* and *SERPINA3* by using a small interfering RNA (siRNA) approach did not reverse the #hMADS-CM-induced inhibition of ADCC (Additional file 10: Figure S7), suggesting that the phenotypic alterations of tumor cells induced by exposure to adipocyte-conditioned medium are likely to be complex.

To assess whether the protection of breast cancer cells by adipocytes was specific to ADCC, we performed 3-(4,5-dimethylthiazol-2-yl)-2,5-diphenyltetrazolium bromide (MTT) cytotoxicity assays with trastuzumab emtansine (T-DM1), an antibody-drug conjugate. We found that #hMADS-CM also protected BT-474 cells from the cytotoxicity mediated by T-DM1 (Additional file 11: Figure S8). Collectively, these data suggest that adipocyte-derived factors rapidly increase the expression of several survival genes that may be involved in cancer resistance to targeted therapies.

### Normal human adipose tissue inhibits the antitumor effect of trastuzumab *in vivo*

**Figure 6:**
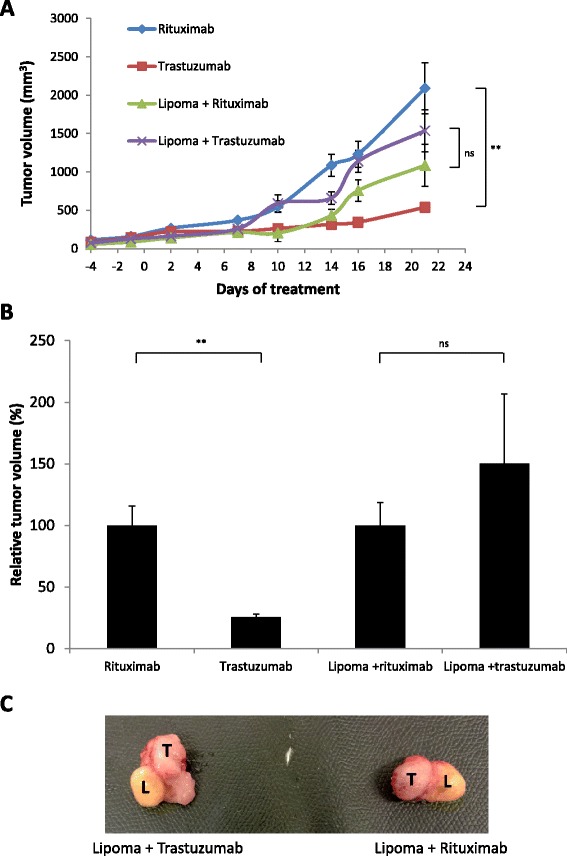
Abdominal adipose tissue inhibits antitumor effect of trastuzumab *in vivo*. **(A)** Tumor growth in BT-474 xenograft mice treated with trastuzumab or rituximab in the presence or absence of lipoma. Median ± standard error of the mean data are shown. **(B)** Normalization of the tumor volumes shown in **(A)** with the tumor volumes of mice treated with rituximab. **(C)** Photos of the tumors in contact with the lipomas taken from mice treated with either trastuzumab (left) or rituximab (right). L, Lipoma; ns, Not significant; T, Tumor. **P < 0.01.

Our results indicate that adipose cells promote cancer resistance to trastuzumab-mediated ADCC *in vitro*. To understand whether adipose tissue can exert the same effect *in vivo*, we conducted the experiment in SCID mice, which have functional macrophages and NK cells [32]. Abdominal adipose tissue obtained from patients undergoing plastic surgery was injected subcutaneously into SCID mice to form a lipoma. BT-474 tumor was grafted next to the lipoma, and mice were treated with trastuzumab or rituximab as a control. The lipoma alone did not grow in the mice (data not shown). As shown in Figure 6A and 6B, trastuzumab inhibited the growth of BT-474 tumor in the absence of lipoma, but not in the presence of lipoma. Moreover, the interface between the lipoma and the tumor was well vascularized (Figure 6C). Therefore, our data strongly suggest that adipose tissue may have an impact on cancer resistance to trastuzumab treatment.

## Discussion

Adipocytes constitute the most abundant cell type in the adipose tissue proximal to breast cancer cells and actively participate in tumor progression through the paracrine secretion of various adipocytokines [33]. Recent studies have suggested that adipocytes promote cancer resistance to chemotherapy and radiotherapy [17,18]. However, the role of these cells in resistance to targeted therapy remains unknown. In this study, using an original coculture system, we investigated the implication of adipocytes and preadipocytes in trastuzumab-mediated ADCC in HER2-overexpressing breast cancer cells.

We demonstrate for the first time that adipocytes and preadipocytes inhibit trastuzumab-mediated tumor lysis by NK cells *in vitro* and that adipose tissue inhibits the antitumor effect of trastuzumab *in vivo*. This highlights the importance of adipose tissue in the resistance of cancer to targeted therapy using monoclonal antibodies. A recent study by Crozier *et al.* showed that increased body mass index (BMI) is associated with shorter disease-free survival in HER2-overexpressing breast cancer patients, although trastuzumab improved clinical outcome, regardless of BMI [34]. However, BMI is only a conventional indicator of the body fat mass and does not reflect the paracrine effect of the local adipose tissue in the microenvironment. Indeed, we observed a reduced antitumor effect of trastuzumab in mice in which the tumor was in direct contact with the lipoma, but not in mice in which the tumor was distantly localized from the lipoma (data not shown). Additionally, we observed an enhancement of the inhibition of ADCC by adipose cells under hypoxic conditions. Because hypoxia is one of the hallmarks of the tumor microenvironment [29] and has also been associated with obesity [35], this latter result underlines the potential clinical relevance of our findings on the impact of adipose tissue on targeted therapy for breast cancer. Furthermore, because adipocytes are hypertrophic with altered functions in obesity [36,37], a comparative study between adipocytes from lean and obese individuals could provide better understanding on the link between obesity and cancer resistance to therapies.

Several studies have pointed out the alteration of NK phenotype or NK functions by the tumor microenvironment [38,39]. Moreover, NK cells have been shown to express receptors for different adipokines, and their cytotoxicity has been found to be modulated by factors such as leptin and adiponectin [40,41]. Here, using NK-92-CD16 cells, we observed a modification of the secretion of IFN-γ, but not any modification of NK cell markers or NK cell cytotoxicity. These results seem to be in contrast to the work by DelaRosa *et al.*, who showed that human ASCs impaired NK cell cytotoxicity and NK cell markers, in particular decreased CD16 expression [42]. However, in DelaRosa’s studies, the NK cells were continuously coincubated with ASCs for 72 hours, whereas in our studies, the incubation times were much shorter (4 hours or 18 hours). Moreover, the use of the NK-92 cell line may be not representative of the situation *in vivo*. Therefore, a possible alteration of NK cells by adipose cells cannot be excluded in our studies. We also found that inhibition of ADCC by adipose tissue was not due to titration or degradation of the therapeutic antibody itself.

Our results suggest that adipose-derived factors reduce the sensitivity of HER2+ breast tumor cells to trastuzumab-mediated ADCC. This is supported by the facts that the ability of adipose cells to inhibit ADCC was variable, depending on the breast tumor cell lines studied, and that BT-474 cells preincubated overnight in #hMADS-CM showed decreased sensitivity to ADCC. We show that exposure of BT-474 cells to #hMADS-CM rapidly upregulated the expression of several genes involved in cell survival. However, the phenotypic alterations of tumor cells induced by exposure to adipocyte-conditioned medium are likely to be complex because downregulation of selected target genes by siRNA did not reverse the adipocyte-induced inhibition of ADCC. Importantly, the protective effect of adipocytes also seemed to apply to other targeted therapies because we observed a protection of BT-474 cells from T-DM1 cytotoxicity in the presence of #hMADS-CM. Likewise, it would be interesting to investigate the impact of adipocytes on the resistance of cancer cells to the adaptive immune system, notably resistance to cytotoxic T cell-mediated cytotoxicity. Further studies are required to better understand the roles of adipose tissue in cancer resistance to therapies.

In an attempt to identify the adipose secreted factors involved in inhibition of ADCC, we tested the ability of known adipocytokines to inhibit ADCC under our experimental conditions. Neither leptin, adiponectin, vaspin, IL-6, IGF-1, autotaxin, TNF-α, nor transforming growth factor β was found to reproduce the inhibition observed with the CM (Additional file 12: Table S3). As the secretomes of adipocytes and preadipocytes are extremely diverse [43], a number of other adipose derived factors could be tested. We hypothesize that the inhibition of ADCC could be mediated by multiple factors that act at the same time on cancer cells.

In a previous study, Dirat *et al.* [44] showed that there is crosstalk between adipocytes and tumor cells and that adipocytes are modified by tumor cells to acquire a typical phenotype, named *cancer-associated adipocytes*, that further enhances tumor cell invasion. In our short-duration coculture, we did not observe any delipidation of adipocytes by tumor cells (data not shown). However, we speculate that the inhibitory effect of ADCC observed with naive adipocytes could be further enhanced by cancer-associated adipocytes.

An important finding in our studies is that abdominal adipose tissue from plastic surgery could inhibit the antitumor effect of trastuzumab *in vivo*. It is worth noting that the biology of adipose tissue can differ substantially, depending on its localization in the human body [45], suggesting that further studies on mammary adipose tissue obtained from mammoplasties could provide more information on the impact of adipose tissue in the breast. Likewise, the impact of adipose tissue on the efficacy of other therapies also requires further investigation. Nonetheless, our data, together with those of others [46,47], raise the question of the safety of lipotransfer in patients undergoing breast reconstruction.

## Conclusions

By using *in vitro* and *in vivo* approaches, we demonstrate that adipocytes and preadipocytes are implicated in the resistance of breast cancer cells to trastuzumab-mediated cytotoxicity. The effects of adipose cells are mediated via the secretion of soluble factors that act on cancer cells to increase their resistance. Further studies are required to identify the adipose-derived factors involved in the crosstalk between adipose stroma and the tumor. This will allow the development of sensitizing strategies to circumvent adipose tissue-mediated resistance to trastuzumab in breast cancer.

## Additional files

Additional file 1: **Supplementary Materials and Methods.**

Additional file 2: Figure S6. Kinetic expression of *GDF15*, *MYC* and *CXCR4* upregulated by #hMADS-CM in BT-474 cells. BT-474 cells were exposed to #hMADS-CM or the control medium for the indicated times. The expression levels of *GDF15*, *MYC* and *CXCR4* were analyzed by RT-qPCR. Fold change indicates the regulation of these genes by #hMADS-CM compared with the control medium. The results shown are mean ± SD values of a duplicate of one experiment representative of three independent experiments.

Additional file 3: Figure S1. #hMADS adipocytes and hMADS preadipocytes inhibit ADCC. ADCC assays on BT-474 cells **(A)** or SK-BR-3 cells and MDA-MB-361 cells **(B)**. Fluorescence intensity of a representative experiment from among at least three independent experiments, each performed in triplicate, is shown in (A). Values are means ± SD of the triplicate. The percentage of cytotoxicity is shown in (B). Values are means ± SD of at least three independent experiments.

Additional file 4: Figure S2. HER2 expression levels in the breast cancer cell lines studied. BT-474 (red lines), MDA-MB-453 (blue lines), SK-BR-3 (violet lines) and MDA-MB-361 (green lines) cells were labeled with anti-HER2 Affibody and analyzed by fluorescence-activated cell sorting (FACS). Dotted lines indicate unstained cells, and solid lines indicate HER2-stained cells. The results shown are representative of three independent experiments.

Additional file 5: Figure S3. Kinetics of ADCC in the presence of adipocyte-conditioned media and effect of proteinase K. **(A)** ADCC assays were performed on BT-474 cells at different kinetic time points in the presence of #hMADS-CM (left) or hMADS-CM (right). The results shown are representative of three independent experiments. **(B)** #hMADS-CM was incubated with 100 μg/ml proteinase K for 1 hour at 37°C. Proteinase K was inactivated by addition of 75 μg/ml phenylmethylsulfonyl fluoride. #hMADS-CM and its control medium were used in ADCC assays. Values are means ± SD of at least three independent experiments.

Additional file 6: Figure S4. hMADS and #hMADS cells do not express FcRs. hMADS and #hMADS cells were labeled with anti-CD16, anti-CD32 or anti-CD64 antibodies; washed; and analyzed by FACS. NK-92-CD16 cells were used as a positive control for CD16 expression, and monocytes were used as a positive control for CD32 and CD64 expression. Dotted red lines indicate unstained cells, and solid green lines indicate the corresponding antibodies. The results shown are representative of three independent experiments.

Additional file 7: Figure S5. #hMADS-CM and hMADS-CM do not modify NK cell viability. NK-92-CD16 cells were preincubated overnight with #hMADS-CM, hMADS-CM or the control media; washed; and counted for viability using trypan blue. Mean ± SD values of three independent experiments are shown.

Additional file 8: Table S1. List of genes up- or downregulated by #hMADS-CM in BT-474 cells.

Additional file 9: Table S2. List of genes up- or downregulated by #hMADS-CM in SK-BR-3 cells.

Additional file 10: Figure S7. Downregulation of *GDF15*, *MYC* and *SERPINA3* by siRNA in ADCC assays. BT-474 cells were transfected with 10 nM scrambled siRNA or siRNA of indicated target genes for 48 hours. At 48 hours posttransfection, gene expression levels of target genes were analyzed by RT-qPCR **(A)** and BT-474 cells were used for ADCC assays **(B)** in the presence of the control medium or #hMADS-CM. The results shown are means ± SD of at least three independent experiments.

Additional file 11: Figure S8. Protection of BT-474 cells by #hMADS-CM from T-DM1. BT-474 cells were exposed to the indicated concentrations of T-DM1 in the presence of the control medium or #hMADS-CM for 72 hours. Cell proliferation was determined by MTT assay. The results shown are representative of three independent experiments.

Additional file 12: Table S3. List of adipocyte-derived factors tested in ADCC assays.

## Abbreviations

ADCC: Antibody-dependent cellular cytotoxicity
ASC: Adipose-derived stem cell
BMI: Body mass index
CM: Conditioned media
DMEM: Dulbecco’s modified Eagle’s medium
ER: Estrogen receptor
FACS: Fluorescence-activated cell sorting
FcR: Fc receptor
FCS: Fetal calf serum
FGF2: Basic fibroblast growth factor
FITC: Fluorescein isothiocyanate
HER2: Human epidermal growth factor receptor 2
hMADS: Human multipotent adipose-derived stem cells
#hMADS: Differentiated human multipotent adipose-derived stem cells
HME: Human mammary epithelial cell
IFN: Interferon
IGF-1: Insulin-like growth factor 1
IL: Interleukin
mTOR: Mammalian target of rapamycin
MTT: 3-(4,5-dimethylthiazol-2-yl)-2,5-diphenyltetrazolium bromide
MUC4: Mucin 4
NK: Natural killer
PTEN: Phosphatase and tensin homolog
qPCR: Quantitative polymerase chain reaction
RT: Reverse transcription
SCID: Severe combined immunodeficiency
siRNA: Small interfering RNA
T-DM1: Trastuzumab emtansine
TNF: Tumor necrosis factor
