# Supplementary material for: Adipose cells promote resistance of breast cancer cells to trastuzumab-mediated antibody-dependent cellular cytotoxicity
Source: Breast Cancer Res. 2015 Apr 24;17(1):57. doi: 10.1186/s13058-015-0569-0 (PMC4482271; doi:10.1186/s13058-015-0569-0)
Supplement: Supplementary file 1 — Supplementary Materials and Methods. [file 13058_2015_569_MOESM1_ESM.doc]

*Induction of hMADS differentiation*

The differentiation of hMADS cells was previously described (1,2). Briefly, hMADS cells were plated at high density (30,000 cells/cm²) in growth medium containing 2.5 ng/mL FGF2. Two days later, the medium was changed without FGF2 and cells were cultured to reach confluence for additional two days. Cells were induced for differentiation in DMEM/F12 medium supplemented with insulin (5 µg/mL), transferrin (10 µg/mL), T3 (0.2 nM), rosiglitazone (1 µM), 3-Isobutyl-1-methylxanthine (IBMX) (100 µM), and dexamethasone (1 µM) (day 0). At day 3, the medium was changed without IBMX nor dexamethasone. At day 12, the medium was replaced and supplemented with 10% FCS. Differentiated hMADS (#hMADS) cells were used at day 14 of differentiation. The differentiation yield was estimated to range between 70 and 80% and was verified by Oil Red O (Sigma) staining, as described previously (3). The conditioned media (CM) from hMADS and #hMADS cells were harvested after centrifugation at 300 g for 5 min and frozen at -20°C before use.

*Retroviral transduction of NK-92 cells*

NK-92, the human NK cell line (4), generously provided by Conkwest (Del Mar, CA), was grown in complete RPMI culture medium. NK-92-CD16 cells were obtained by transduction of pMX/CD16 plasmid (5) using retroviral supernatant. Transient retroviral supernatants were produced by CaCl_2_ precipitation (Invitrogen) with 15 µg pMX/CD16 plasmid using Phoenix-Ampho cells. The conditioned medium was collected 24-48 h post-transfection, filtered through 0.45-µm pore-size filters and kept at -80°C until use. The viral titer was determined by the transduction of Jurkat T cells (10^6^ cells per well in 6-well plates) with serial dilutions. Retroviral supernatant titers were typically 1-5*10^5^ IU (Infectious Units)/ml. The NK-92 cell line was seeded at 10^6^ cells into 6-well plates and exposed to 2*2 ml of retroviral supernatant by spinoculation (2400 g, 1.5 h, 32°C) in the presence of 4 µg/ml polybrene (Sigma, St Quentin Fallavier, France) to generate the NK-92-CD16 cells. The culture medium was changed 24 h post-infection. Transduction efficiencies were assessed 5 days later by flow cytometry after staining with the CD16 PE-conjugated mouse anti-CD16 antibody (clone 3G8) (Beckman Coulter, Roissy, France). NK-92-CD16 cells were selected for higher CD16 expression by cell sorter flow cytometry using anti-CD16 antibody (Beckman Coulter, Roissy, France). Purity was >95%.

*Antibodies*

The antibodies used were purchased from BD Biosciences (anti-CD16 FITC clone 3G8, anti-CD25 FITC clone 2A3, anti-CD56 V450 clone B159, anti-CD69 PE clone FN50, anti-CD107a PE clone H4A3), Miltenyi (anti-CD159a (NKG2A) FITC clone REA110, anti-CD314 (NKG2D) APC clone BAT221, anti-CD336 (NKp44) APC clone 2.29, and anti-CD337 (NKp30) PE clone AF29-4D12). Anti-ErbB2 (HER2) affibody FITC was purchased from Abcam.

1. Rodriguez A-M, Pisani D, Dechesne CA, Turc-Carel C, Kurzenne J-Y, Wdziekonski B, et al. Transplantation of a multipotent cell population from human adipose tissue induces dystrophin expression in the immunocompetent mdx mouse. J Exp Med. 2005 May 2;201(9):1397–405.

2. Zaragosi L-E, Ailhaud G, Dani C. Autocrine fibroblast growth factor 2 signaling is critical for self-renewal of human multipotent adipose-derived stem cells. Stem Cells Dayt Ohio. 2006 Nov;24(11):2412–9.

3. Abderrahim-Ferkoune A, Bezy O, Astri-Roques S, Elabd C, Ailhaud G, Amri E-Z. Transdifferentiation of preadipose cells into smooth muscle-like cells: role of aortic carboxypeptidase-like protein. Exp Cell Res. 2004 Feb 15;293(2):219–28.

4. Gong JH, Maki G, Klingemann HG. Characterization of a human cell line (NK-92) with phenotypical and functional characteristics of activated natural killer cells. Leukemia. 1994 Apr;8(4):652–8.

5. Clémenceau B, Congy-Jolivet N, Gallot G, Vivien R, Gaschet J, Thibault G, et al. Antibody-dependent cellular cytotoxicity (ADCC) is mediated by genetically modified antigen-specific human T lymphocytes. Blood. 2006 Jun 15;107(12):4669–77.
