# Supplementary material for: Adipose cells promote resistance of breast cancer cells to trastuzumab-mediated antibody-dependent cellular cytotoxicity
Source: Breast Cancer Res. 2015 Apr 24;17(1):57. doi: 10.1186/s13058-015-0569-0 (PMC4482271; doi:10.1186/s13058-015-0569-0)
Supplement: Supplementary file 2 — Kinetic expression of GDF15, MYC and CXCR4 upregulated by #hMADS-CM in BT-474 cells. BT-474 cells were exposed to #hMADS-CM or the control medium for the indicated times. The expression levels of GDF15, MYC and CXCR4 were analyzed by RT-qPCR. Fold change indicates the regulation of these genes by #hMADS-CM compared with the control medium. The results shown are mean ± SD values of a duplicate of one experiment representative of three independent experiments. [file 13058_2015_569_MOESM2_ESM.docx]

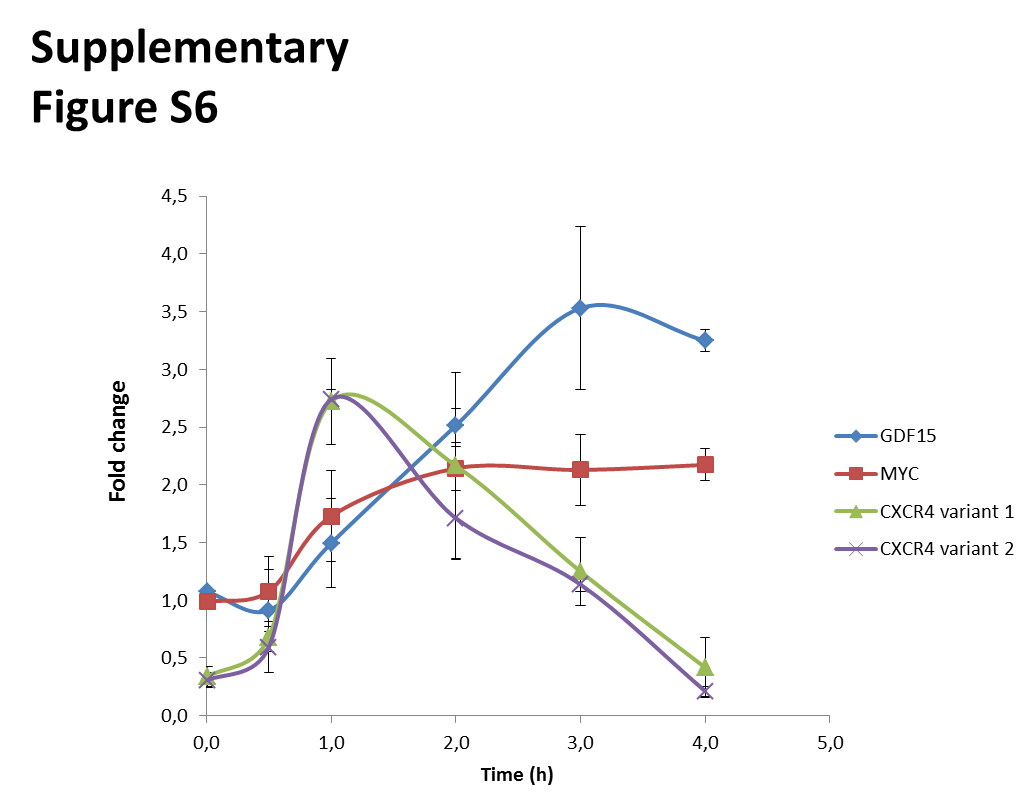


**Fig. S6. Kinetic expression of GDF15, MYC and CXCR4 up-regulated by #hMADS-CM in BT474 cells.** BT474 cells were exposed to #hMADS-CM or the control medium for indicated times. The expression of *GDF15*, *MYC* and *CXCR4* were analyzed by RT-qPCR. Fold change indicates the regulation of these genes by #hMADS-CM compared to the control medium. Results shown are means ± SD of a duplicate of one experiment representative of three independent experiments.
