# Supplementary material for: Adipose cells promote resistance of breast cancer cells to trastuzumab-mediated antibody-dependent cellular cytotoxicity
Source: Breast Cancer Res. 2015 Apr 24;17(1):57. doi: 10.1186/s13058-015-0569-0 (PMC4482271; doi:10.1186/s13058-015-0569-0)
Supplement: Supplementary file 3 — #hMADS adipocytes and hMADS preadipocytes inhibit ADCC. ADCC assays on BT-474 cells (A) or SK-BR-3 cells and MDA-MB-361 cells (B). Fluorescence intensity of a representative experiment from among at least three independent experiments, each performed in triplicate, is shown in (A). Values are means ± SD of the triplicate. The percentage of cytotoxicity is shown in (B). Values are means ± SD of at least three independent experiments. [file 13058_2015_569_MOESM3_ESM.docx]

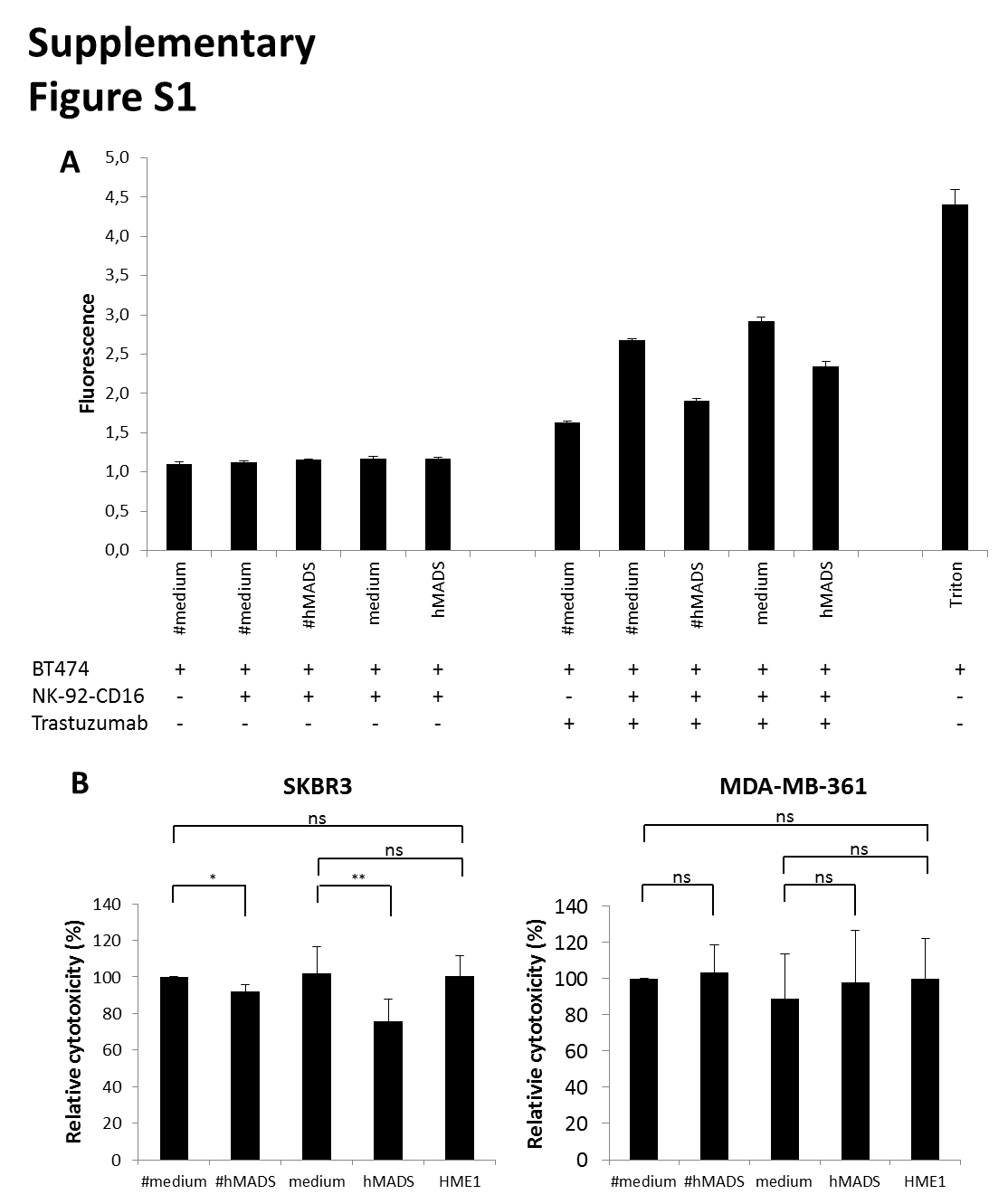


**Fig. S1. #hMADS adipocytes and hMADS preadipocytes inhibit ADCC**. A) BT474 cells were labeled with 12.5 µM calcein and incubated with 1 µg/mL trastuzumab and NK-92-CD16 cells at effector to target (E:T) ratio = 5:1, in the presence of the control media, #hMADS, hMADS cells. After 4 h of incubation, the fluorescence signals of calcein released into the supernatant due to the lysis of target BT474 cells were measured. Representative experiment of at least 3 independent experiments, each performed in triplicate, is shown. Values are means ± SD of the triplicate. B) Same as in A), but ADCC assays were performed on SKBR3 and MDA-MB-361 cells. The percentage of cytotoxicity was calculated. Values are means ± SD of at least three independent experiments. *p<0.05; **p<0.01; ns: not significant.
