# Supplementary material for: Adipose cells promote resistance of breast cancer cells to trastuzumab-mediated antibody-dependent cellular cytotoxicity
Source: Breast Cancer Res. 2015 Apr 24;17(1):57. doi: 10.1186/s13058-015-0569-0 (PMC4482271; doi:10.1186/s13058-015-0569-0)
Supplement: Supplementary file 4 — HER2 expression levels in the breast cancer cell lines studied. BT-474 (red lines), MDA-MB-453 (blue lines), SK-BR-3 (violet lines) and MDA-MB-361 (green lines) cells were labeled with anti-HER2 Affibody and analyzed by fluorescence-activated cell sorting (FACS). Dotted lines indicate unstained cells, and solid lines indicate HER2-stained cells. The results shown are representative of three independent experiments. [file 13058_2015_569_MOESM4_ESM.docx]

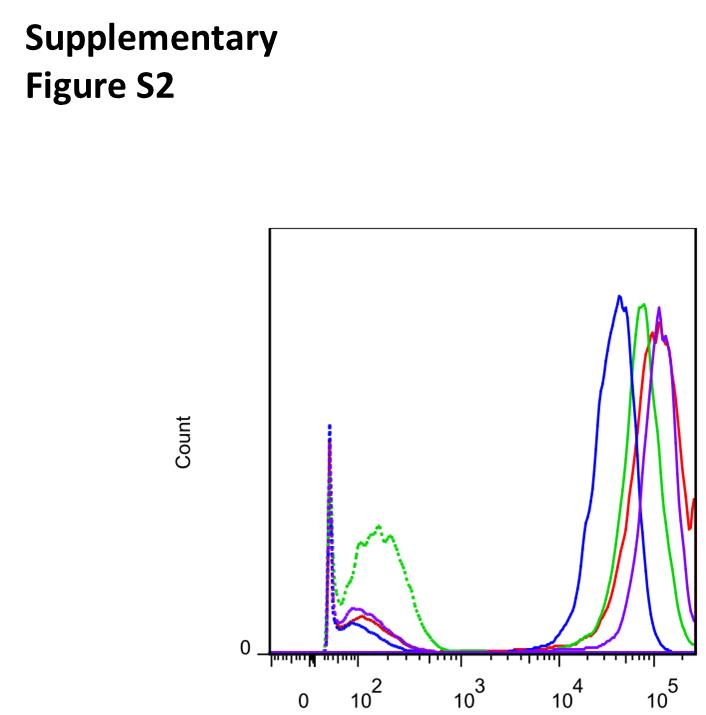


**Fig. S2. HER2 expression levels in the breast cancer cell lines studied.** BT474 (red lines), MDA-MB-453 (blue lines), SKBR3 (violet lines) and MDA-MB-361 (green lines) cells were labeled with anti-HER2 affibody and analyzed by FACS. Dotted lines indicate unstained cells and solid lines indicate HER2-stained cells. Results representative of 3 independent experiments are shown.
