# Supplementary material for: Adipose cells promote resistance of breast cancer cells to trastuzumab-mediated antibody-dependent cellular cytotoxicity
Source: Breast Cancer Res. 2015 Apr 24;17(1):57. doi: 10.1186/s13058-015-0569-0 (PMC4482271; doi:10.1186/s13058-015-0569-0)
Supplement: Supplementary file 5 — Kinetics of ADCC in the presence of adipocyte-conditioned media and effect of proteinase K. (A) ADCC assays were performed on BT-474 cells at different kinetic time points in the presence of #hMADS-CM (left) or hMADS-CM (right). The results shown are representative of three independent experiments. (B) #hMADS-CM was incubated with 100 μg/ml proteinase K for 1 hour at 37°C. Proteinase K was inactivated by addition of 75 μg/ml phenylmethylsulfonyl fluoride. #hMADS-CM and its control medium were used in ADCC assays. Values are means ± SD of at least three independent experiments. [file 13058_2015_569_MOESM5_ESM.docx]

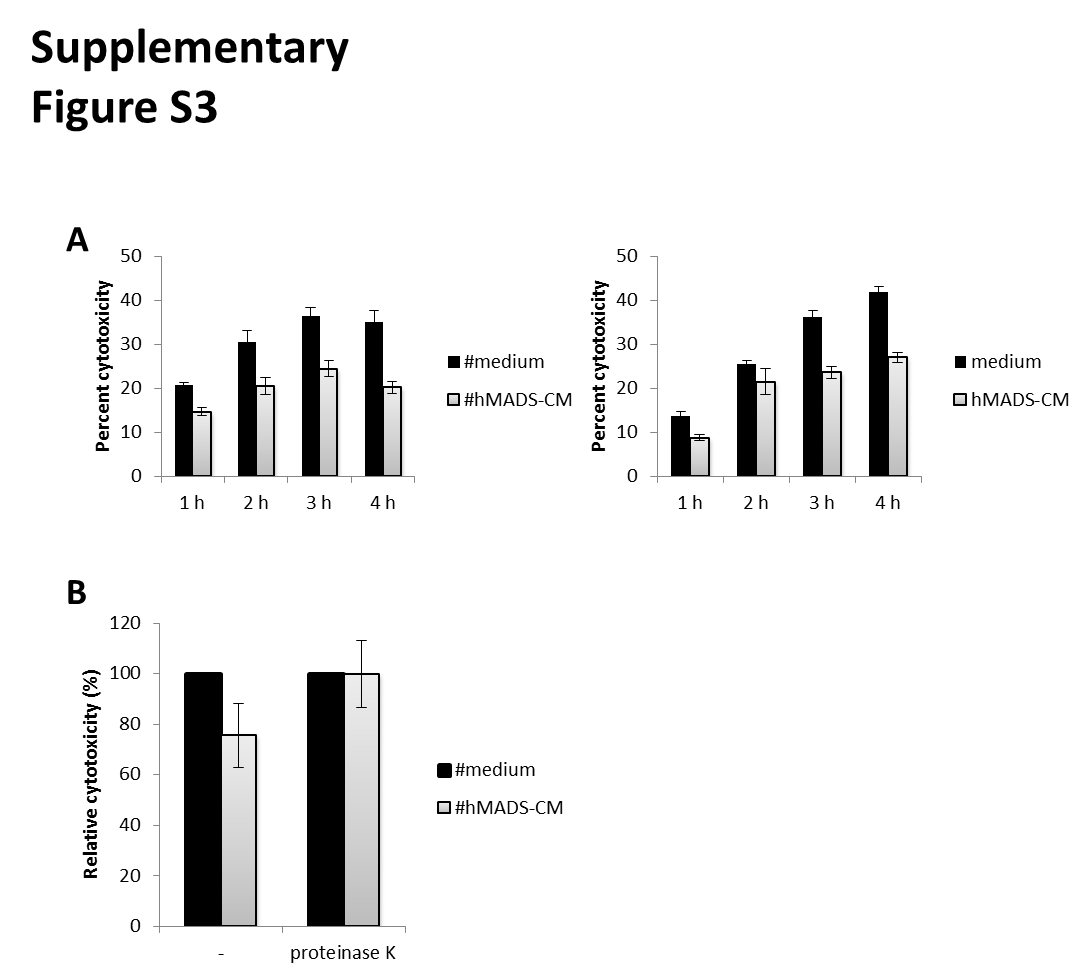


**Fig. S3. Kinetics of ADCC in the presence of adipose conditioned media and effect of proteinase K.** A) ADCC assay was performed on BT474 cells at different kinetic time points, in the presence of #hMADS-CM (left) or hMADS-CM (right). Figure presents the results of a representative experiment out of three with similar results, each performed in quadruplicate. Values are means ± SD of the quadruplicate. B) #hMADS-CM was incubated with 100 µg/mL proteinase K for 1 h at 37°C. Proteinase K was inactivated by addition of 75 µg/mL PMSF. #hMADS-CM and its control medium were used in ADCC assays. Values are means ± SD of at least three independent experiments.
