# Supplementary material for: Adipose cells promote resistance of breast cancer cells to trastuzumab-mediated antibody-dependent cellular cytotoxicity
Source: Breast Cancer Res. 2015 Apr 24;17(1):57. doi: 10.1186/s13058-015-0569-0 (PMC4482271; doi:10.1186/s13058-015-0569-0)
Supplement: Supplementary file 6 — hMADS and #hMADS cells do not express FcRs. hMADS and #hMADS cells were labeled with anti-CD16, anti-CD32 or anti-CD64 antibodies; washed; and analyzed by FACS. NK-92-CD16 cells were used as a positive control for CD16 expression, and monocytes were used as a positive control for CD32 and CD64 expression. Dotted red lines indicate unstained cells, and solid green lines indicate the corresponding antibodies. The results shown are representative of three independent experiments. [file 13058_2015_569_MOESM6_ESM.docx]

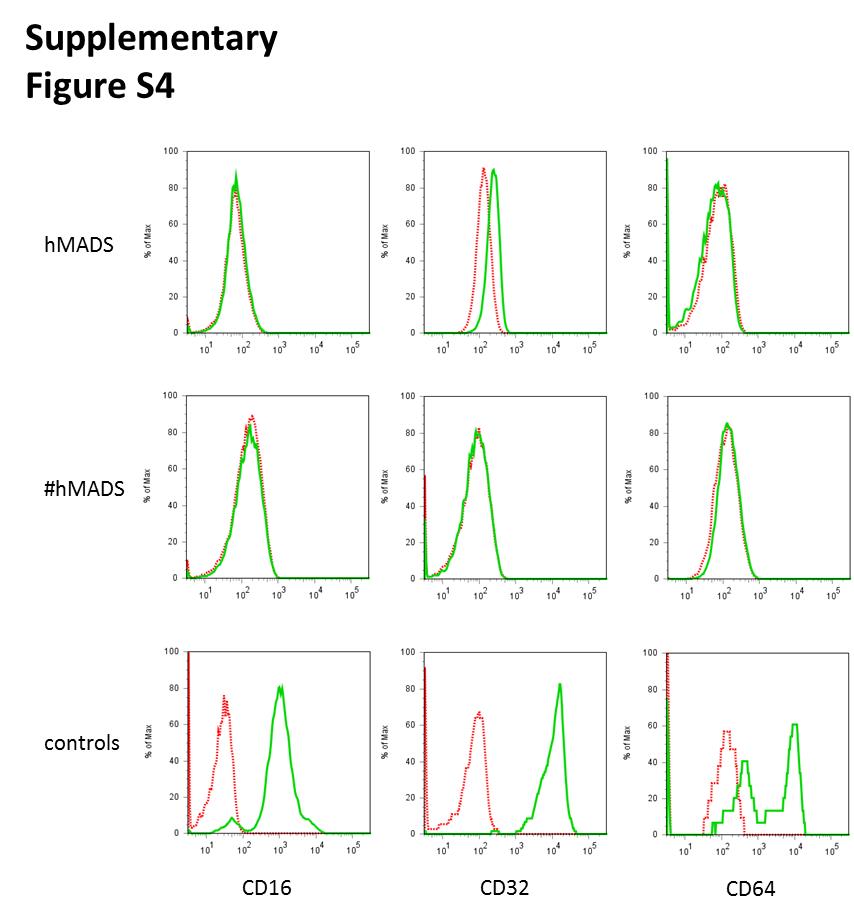


**Supplementary Figure S4. hMADS and #hMADS cells do not express FcR.** hMADS and #hMADS cells were labeled with anti-CD16 or anti-CD32 or anti-CD64 antibodies, washed and analyzed by FACS. NK-92-CD16 cells were used as positive control of CD16 expression; monocytes were used as positive control of CD32 and CD64 expression. Dotted red lines indicate unstained cells and solid green lines indicate corresponding antibodies. Results representative of 3 independent experiments are shown.
