# Supplementary material for: Adipose cells promote resistance of breast cancer cells to trastuzumab-mediated antibody-dependent cellular cytotoxicity
Source: Breast Cancer Res. 2015 Apr 24;17(1):57. doi: 10.1186/s13058-015-0569-0 (PMC4482271; doi:10.1186/s13058-015-0569-0)
Supplement: Supplementary file 7 — #hMADS-CM and hMADS-CM do not modify NK cell viability. NK-92-CD16 cells were preincubated overnight with #hMADS-CM, hMADS-CM or the control media; washed; and counted for viability using trypan blue. Mean ± SD values of three independent experiments are shown. [file 13058_2015_569_MOESM7_ESM.docx]

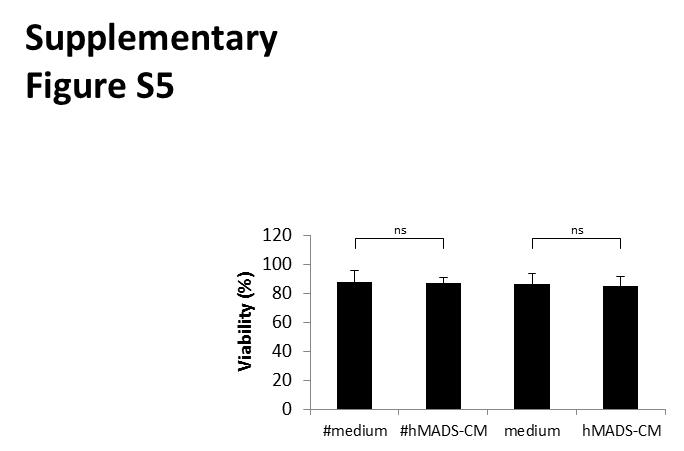


**Supplementary Figure S5. #hMADS-CM and hMADS-CM do not modify NK cell viability.** NK-92-CD16 cells were preincubated overnight with #hMADS-CM or hMADS-CM or the control media, washed and counted for viability using trypan blue. Means ± SD of 3 independent experiments are shown. Ns: not significant.
