# Supplementary material for: Adipose cells promote resistance of breast cancer cells to trastuzumab-mediated antibody-dependent cellular cytotoxicity
Source: Breast Cancer Res. 2015 Apr 24;17(1):57. doi: 10.1186/s13058-015-0569-0 (PMC4482271; doi:10.1186/s13058-015-0569-0)
Supplement: Supplementary file 8 — List of genes up- or downregulated by #hMADS-CM in BT-474 cells. [file 13058_2015_569_MOESM8_ESM.docx]

**Supplementary Table 1. List of genes up- or down-regulated by #hMADS-CM in BT474 cells**

| **Gene name** | **Accession No** | **Fold change** | **p value** | **Classification** |
| --- | --- | --- | --- | --- |
| ADM | NM_001124.1 | 2.54 | 2.66E-02 | Hormone activity |
| GDF15 | NM_004864.1 | 2.52 | 5.28E-02 | Growth factor, cytokine activity |
| MYC | NM_002467.3 | 2.03 | 7.20E-03 | Transcription |
| SERPINA3 | NM_001085.4 | 1.91 | 9.18E-03 | Serine protease inhibition |
| PMAIP1 | NM_021127.1 | 1.78 | 8.97E-03 | Pro-apoptosis |
| NCOA7 | NM_181782.2 | 1.76 | 6.23E-02 | Nuclear receptor coactivator |
| EGR1 | NM_001964.2 | 1.74 | 1.13E-01 | Transcription |
| DKK1 | NM_012242.2 | 1.73 | 6.83E-02 | Wnt pathway inhibition |
| NLF2  DUSP10  IRF1  LOC402644  ZNF703  EGR3  TNFAIP8  MYADM  CXCR4  BCL6  CEBPD  RGS16  IER3  GABARAP  HSPB8  BHLHB2  LOC641848  LOC392285  GADD45G  LOC653702  DUSP6  PIM3  RPLP1  RPS26P10  FHL2  KRT18P28  LOC730288  LOC642989  LOC728115  LOC644037  LOC100130154  SLC25A25  LOC730029  LOC100132199  SPRY4  ELF3  LOC644330  LOC727821  PHF23  GPRC5A  PTMA  HSPE1  ZFP36L2  ERRFI1  LOC100129028  UQCRH  LOC100134504  DUSP5  RBMX2  ERF  LOC440595  RARA  LOC441073  LOC388076  FRMD6  LOC728843  HMGB1  LOC100127922  LOC728026  LOC644937  WSB2  LOC649946  FOSB  LOC729255  RAB30  LOC402251  LOC100128899  KPNA2  HSPA4  KCNK1  LOC128192  ANP32A  LOC645236  MID1IP1  LOC729687  IER5L  CDC42EP2  FAM89A  LOC649839  LOC728590  LOC100131572  MCL1  HNRPC  C7orf28B  LOC649873  LOC347376  ANXA2P1  MAFF  NOP56  LOC728484  PDLIM3  RPS28  LMCD1  ZFP36L1  RNF39  TGIF1  GPKOW  FTHL11  PSMC6  SHISA2  LRRC59  AK3L1  LOC441550  TACSTD1  PRAGMIN  LOC645630  LOC654074  LOC100131989  LOC651919  LOC389787  CD59  LOC732165  ZFP36  TEAD3  LOC388344  ZNF302  NUPR1  GPER  ARL6IP4  CCNG2  LOC643031  DDIT4  LOC100129882  MIR638  SMAD6 | NM_001007595.1  NM_144729.1  NM_002198.1  XM_938297.1  NM_025069.1  NM_004430.2  NM_001077654.1  NM_138373.3  NM_001008540.1  NM_001706.2  NM_005195.2  NM_002928.2  NM_052815.1  NM_007278.1  NM_014365.2  NM_003670.1  XM_935588.1  XR_017134.2  NM_006705.2  XM_938789.1  NM_022652.2  NM_001001852.2  NM_001003.2  XM_376787.3  NM_001450.3  XR_017689.1  XM_001126276.1  XM_926370.1  XR_038319.1  XR_038280.1  XM_001717333.1  NM_052901.2  XM_001724847.1  XR_039723.1  NM_030964.2  NM_004433.3  XM_934365.1  XR_037166.1  NM_024297.1  NM_003979.3  NM_002823.2  NM_002157.1  NM_006887.3  NM_018948.2  XM_001722134.1  NM_006004.1  XM_001725687.1  NM_004419.3  NM_016024.1  NM_006494.1  XR_038356.1  NM_001024809.2  XR_018937.2  XM_001722259.1  NM_152330.2  XR_015391.1  NM_002128.3  XR_038410.1  XM_001126659.2  XM_941010.3  NM_018639.3  NR_003040.1  NM_006732.1  XR_016047.1  NM_014488.3  XM_377933.3  XR_039503.1  NM_002266.2  NM_002154.3  NM_002245.2  XM_001725191.1  NM_006305.2  XM_928275.1  NM_021242.3  XM_001725257.1  NM_203434.1  NM_006779.2  XM_939093.1  XM_938903.3  XM_001131826.2  XM_001725183.1  NM_021960.3  NM_031314.1  NM_198097.1  XR_037451.1  XM_937928.1  NR_001562.1  NM_012323.2  XM_936090.1  XR_015270.2  NM_014476.1  NM_001031.4  NM_014583.2  NM_004926.2  NM_170770.1  NM_170695.2  NM_015698.3  NR_002204.1  NM_002806.2  NM_001007538.1  NM_018509.2  NM_001005353.1  XR_038097.1  NM_002354.1  NM_001080826.1  XR_039656.1  XM_941635.1  XM_001725073.1  XM_941189.1  XM_497072.2  NM_203329.1  XM_934933.1  NM_003407.1  NM_003214.2  XM_371023.4  NM_018443.2  NM_001042483.1  NM_001031682.1  NM_001002251.1  NM_004354.1  XM_926402.1  NM_019058.2  XM_001716882.1  NR_030368.1  NM_005585.2 | 1.69  1.68  1.67  1.65  1.64  1.57  1.54  1.54  1.53  1.53  1.53  1.52  1.52  1.52  1.52  1.49  1.48  1.48  1.47  1.47  1.46  1.46  1.46  1.45  1.44  1.43  1.43  1.43  1.43  1.42  1.42  1.42  1.41  1.41  1.40  1.40  1.40  1.40  1.39  1.39  1.39  1.39  1.39  1.38  1.38  1.38  1.38  1.38  1.37  1.37  1.37  1.37  1.36  1.36  1.36  1.36  1.36  1.36  1.35  1.35  1.35  1.35  1.35  1.35  1.35  1.35  1.35  1.35  1.35  1.35  1.35  1.34  1.34  1.34  1.34  1.34  1.34  1.34  1.34  1.34  1.34  1.34  1.33  1.33  1.33  1.33  1.33  1.33  1.32  1.32  1.32  1.32  1.32  1.32  1.32  1.32  1.32  1.32  1.31  1.31  1.31  1.31  1.31  1.31  1.31  1.31  1.31  1.31  1.31  1.31  1.30  1.30  1.30  1.30  1.30  0.77  0.77  0.76  0.76  0.75  0.74  0.72  0.71  0.62  0.61 | 1.45E-02  1.84E-04  6.21E-03  4.73E-02  1.76E-02  1.29E-02  1.52E-02  7.10E-02  1.45E-02  1.09E-02  1.29E-02  3.82E-03  1.13E-01  1.59E-01  8.13E-03  1.36E-02  7.59E-02  1.43E-02  2.04E-02  2.77E-02  1.57E-03  8.22E-02  1.58E-01  8.98E-02  1.36E-02  4.58E-02  6.62E-02  4.71E-02  5.49E-02  7.23E-02  1.52E-01  1.70E-02  5.27E-02  6.38E-02  1.80E-02  1.20E-03  3.09E-02  7.09E-02  1.11E-02  3.86E-02  1.33E-01  5.36E-02  5.60E-02  4.74E-02  5.56E-02  1.07E-01  1.17E-01  5.44E-02  6.19E-02  5.31E-02  5.23E-02  1.16E-04  1.41E-01  1.11E-01  2.12E-02  1.25E-01  1.35E-02  7.82E-02  9.07E-02  2.31E-02  2.37E-02  1.41E-02  1.79E-01  1.05E-01  7.15E-03  7.48E-03  6.37E-02  1.57E-01  1.14E-02  2.43E-02  1.30E-01  5.45E-02  7.67E-02  3.23E-02  6.79E-02  1.25E-01  2.06E-03  1.57E-04  1.07E-01  9.42E-02  1.90E-01  4.72E-02  7.99E-02  4.82E-02  8.44E-02  1.75E-02  3.38E-02  1.23E-02  1.83E-02  4.70E-02  7.44E-03  2.45E-02  2.81E-02  1.04E-01  1.65E-02  1.12E-01  2.72E-02  9.97E-02  4.07E-02  7.14E-02  7.15E-02  9.26E-02  7.55E-02  1.54E-02  1.65E-02  1.64E-01  1.12E-01  4.63E-02  1.17E-01  7.07E-02  2.65E-02  7.34E-02  8.42E-02  4.12E-02  1.04E-01  1.07E-02  4.18E-02  2.94E-03  9.06E-04  7.31E-02  1.55E-01  2.58E-02  2.44E-01  3.81E-01  1.38E-05 | Transcription  MAPK pathway inhibition  Transcription  Transcription  Transcription  Anti-apoptosis  Chemokine receptor  Transcription  Transcription  Inhibition of G protein signaling  Anti-apoptosis  Apoptosis. autophagy  Chaperone activity  Transcription  Growth. apoptosis  MAPK pathway inhibition  Anti-apoptosis  Translation  Pseudogene  Transcription  Pseudogene  Mitochondrial solute carrier  MAPK pathway inhibition  Transcription  G protein signaling  Immune function  Chaperone activity  Transcription  Inhibition of EGFR signaling  Mitochondrial respiratory chain  MAPK pathway inhibition  RNA binding activity  Transcription  Transcription  Pseudogene  Transcription. cytokine activity  Ubiquitination  Transcription  Intracellular membrane trafficking  Nuclear protein import  Potassium channel  Pseudogene  Transcription  Regulation of lipogenesis  Organization of actin cytoskeleton  Apoptosis  Translation  Pseudogene  Pseudogene  Pseudogene  Transcription  Translation  Organization of actin cytoskeleton  Translation  Transcription  Transcription  Transcription  Nucleic acid binding activity  Pseudogene  Proteasome activity  Inhibition of FGF and Wnt signaling  Nuclear protein import  GTP:ATP phosphotransferase activity  Adhesion  Tyrosine kinase activity  Inhibitor of complement membrane attack complex  Transcription  Transcription  Pseudogene  Transcription  Transcription. anti-apoptosis  G-protein coupled estrogen receptor  Cell growth. cell cycle inhibition  mTORC1 inhibition  microRNA  Anti-inflammation |
| CYP1B1 | NM_000104.2 | 0.55 | 3.98E-02 | Metabolism |
| ID2 | NM_002166.4 | 0.47 | 2.94E-04 | Transcription |
| CYP1A1 | NM_000499.2 | 0.33 | 2.87E-03 | Metabolism |
| ID1 | NM_181353.1 | 0.18 | 2.06E-07 | Transcription |
| ID3 | NM_002167.2 | 0.16 | 2.83E-06 | Transcription |

Only genes with more than 1.3-fold changes are shown
