# Supplementary material for: Adipose cells promote resistance of breast cancer cells to trastuzumab-mediated antibody-dependent cellular cytotoxicity
Source: Breast Cancer Res. 2015 Apr 24;17(1):57. doi: 10.1186/s13058-015-0569-0 (PMC4482271; doi:10.1186/s13058-015-0569-0)
Supplement: Supplementary file 9 — List of genes up- or downregulated by #hMADS-CM in SK-BR-3 cells. [file 13058_2015_569_MOESM9_ESM.docx]

**Supplementary Table 2. List of genes up- or down-regulated by #hMADS-CM in SKBR3 cells**

| **Gene name** | **Accession No** | **Fold change** | | **p value** | | **Classification** | |
| --- | --- | --- | --- | --- | --- | --- | --- |
| FGG | NM_000509.4 | 7.33 | 7.83E-04 | | Extracellular matrix composition | |  |
| CEBPD | NM_005195.2 | 5.12 | 3.40E-04 | | Transcription | |  |
| CCL2 | NM_002982.3 | 4.68 | 1.98E-03 | | Cytokine activity | |  |
| FGB | NM_005141.2 | 4.32 | 1.17E-04 | | Extracellular matrix composition | |  |
| NCOA7 | NM_181782.2 | 3.62 | 7.92E-05 | | Nuclear receptor coactivator activity | |  |
| STEAP4 | NM_024636.1 | 2.89 | 1.22E-03 | | Oxidoreductase activity | |  |
| TMEM2 | NM_013390.1 | 2.76 | 1.57E-04 | | Morphogenesis | |  |
| IRF1 | NM_002198.1 | 2.68 | 2.00E-03 | | Transcription | |  |
| PHLDA1 | NM_007350.2 | 2.63 | 5.64E-03 | | Anti-apoptosis | |  |
| CDKN1A | NM_078467.1 | 2.59 | 5.74E-04 | | Cell cycle regulation | |  |
| NLF2 | NM_001007595.1 | 2.52 | 4.65E-04 | | Inflammation, adhesion | |  |
| RGS16 | NM_002928.2 | 2.52 | 5.31E-03 | | Signal transduction | |  |
| TFPI2 | NM_006528.2 | 2.39 | 6.80E-04 | | Serine protease inhibition | |  |
| SOCS2 | NM_003877.3 | 2.33 | 3.20E-04 | | Signal transduction | |  |
| SERPINA3 | NM_001085.4 | 2.33 | 3.11E-04 | | Serine protease inhibition | |  |
| RASL11A | NM_206827.1 | 2.29 | 2.87E-04 | | GTPase activity | |  |
| ADM | NM_001124.1 | 2.28 | 2.14E-03 | | Hormone activity | |  |
| PPAP2B | NM_003713.3 | 2.27 | 1.62E-03 | | Lipid phosphatase activity | |  |
| LRG1 | NM_052972.2 | 2.26 | 2.27E-04 | | Signal transduction, adhesion | |  |
| UGCG | NM_003358.1 | 2.24 | 4.52E-04 | | Ceramide glucosyltransferase activity | |  |
| FAM83A | NM_032899.4 | 2.23 | 2.15E-04 | | Signal transduction | |  |
| GPRC5A | NM_003979.3 | 2.22 | 5.98E-03 | | Signal transduction | |  |
| IER5 | NM_016545.3 | 2.21 | 8.41E-04 | | Transcription | |  |
| F2RL1 | NM_005242.3 | 2.21 | 4.31E-03 | | Coagulation | |  |
| ZFP36L2 | NM_006887.3 | 2.18 | 3.82E-04 | | Transcription | |  |
| FHL2 | NM_001450.3 | 2.15 | 1.10E-04 | | Transcription | |  |
| TM4SF1 | NM_014220.2 | 2.11 | 5.54E-04 | | Signal transduction | |  |
| CEBPB | NM_005194.2 | 2.11 | 1.22E-03 | | Transcription | |  |
| RCAN1 | NM_203418.1 | 2.08 | 6.18E-04 | | Transcription | |  |
| BCL3 | NM_005178.2 | 2.07 | 8.23E-05 | | Transcription | |  |
| SOX9 | NM_000346.2 | 2.07 | 6.67E-05 | | Transcription | |  |
| C17ORF96 | NM_001130677.1 | 2.04 | 2.53E-03 | |  | |  |
| CITED4 | NM_133467.2 | 2.04 | 4.69E-04 | | Transcription | |  |
| GPR37 | NM_005302.2 | 2.03 | 1.93E-04 | | Signal transduction | |  |
| HS.543887 | Hs.543887 | 2.02 | 1.22E-01 | |  | |  |
| ETS2 | NM_005239.4 | 2.01 | 1.47E-06 | | Transcription | |  |
| EFNB2 | NM_004093.2 | 2.00 | 2.21E-04 | | Ephrin receptor | |  |
| B4GALT5 | NM_004776.2 | 1.99 | 1.49E-04 | | Galactosyltransferase activity | |  |
| ATF3 | NM_001040619.1 | 1.99 | 1.04E-02 | | Transcription | |  |
| MAFF | NM_012323.2 | 1.97 | 7.49E-04 | | Transcription | |  |
| P2RY6 | NM_176797.1 | 1.97 | 2.37E-04 | | Pyrimidinergic receptor | |  |
| F2RL1 | NM_005242.3 | 1.96 | 3.47E-03 | |  | |  |
| SPRY4 | NM_030964.2 | 1.94 | 1.61E-04 | | Signal transduction | |  |
| NAMPT | NM_182790.1 | 1.93 | 3.57E-03 | | Metabolism | |  |
| CDC42EP2 | NM_006779.2 | 1.92 | 2.20E-03 | | Rho GTPase activator activity | |  |
| ZFP36 | NM_003407.1 | 1.91 | 4.35E-03 | | Transcription | |  |
| TSC22D1 | NM_006022.2 | 1.91 | 1.66E-03 | | Transcription | |  |
| LOC100129882 | XM_001716882.1 | 1.90 | 1.75E-01 | |  | |  |
| OBFC2A | NM_022837.1 | 1.89 | 2.26E-03 | | RNA binding, single-stranded DNA binding | |  |
| SLCO2A1 | NM_005630.1 | 1.88 | 1.14E-02 | | prostaglandin transporter | |  |
| CRISPLD2 | NM_031476.1 | 1.87 | 1.30E-03 | | Heparin binding | |  |
| IER5L | NM_203434.1 | 1.86 | 3.45E-03 | | Transcription | |  |
| PFKFB3 | NM_004566.2 | 1.86 | 3.28E-03 | | Metabolism | |  |
| FAM46C | NM_017709.2 | 1.83 | 1.36E-03 | |  | |  |
| RND1 | NM_014470.2 | 1.83 | 8.74E-03 | | Rho GTPase activator activity | |  |
| KIAA0247 | NM_014734.2 | 1.82 | 7.33E-05 | | Cell cycle regulation | |  |
| LDLR | NM_000527.2 | 1.80 | 1.23E-04 | | Low density lipoprotein receptor | |  |
| MAT2A | NM_005911.4 | 1.79 | 3.90E-03 | | Methionine adenosyltransferase activity | |  |
| BHLHB2 | NM_003670.1 | 1.78 | 1.62E-04 | | Transcription | |  |
| RCAN1 | NM_203417.1 | 1.77 | 6.13E-03 | | Transcription | |  |
| YPEL2 | NM_001005404.3 | 1.75 | 1.09E-05 | |  | |  |
| NFIL3 | NM_005384.2 | 1.74 | 5.74E-04 | | Transcription | |  |
| ARID5B | NM_032199.1 | 1.74 | 4.63E-04 | | Transcription | |  |
| IL20 | NM_018724.3 | 1.73 | 2.97E-03 | |  | |  |
| IL1RL1 | NM_003856.2 | 1.71 | 9.26E-03 | | Inflammation | |  |
| CD14 | NM_001040021.1 | 1.70 | 2.63E-04 | | Inflammation | |  |
| TRIB2  DCUN1D3  VAV3  TMEM51  ETS1  PLAUR  IER3  ELL  LBP  SOCS2  FOS  RDH10  BCL6  TRIB1  WDR91  ANKRD22  MID1IP1  ANKRD57  RARA  DKFZp761P0423  PRAGMIN  PHLDB2  BATF  TSC22D1  TNFRSF10A  TMEM173  TUBB3  JUNB  C20orf54  KCNS3  MCL1  ZNF281  IL24  FVT1  BAG3  SAT1  RNF145  CASP4  SGK1  STOM  CHORDC1  IL1R1  DUSP5  P2RY6  SGK  SC4MOL  C7orf40  TICAM1  ANXA1  GSDMC  PRRG1  RNF19A  PHLDB2  PRSS22  KRT17  ITPRIP  SERPINB1  SLC41A1  F3  CHSY1  KCTD6  MAK16  EPHA2  ZNF800  EMP1  GFOD2  RRAGC  FVT1  ERRFI1  UTP23  AUTS2  LOC100134424  IRF2BP2  SH3RF1  GCNT2  TNIP3  AMD1  CCL20  LOC728931  PUS3  DTX2  FAM60A  UTP3  RAB31  SPRY1  IL4R  C3orf52  TOP1P2  ICAM1  NXT1  MARS2  CHST15  SLC35E1  LOC401805  RPS26P10  KIAA1949  KCNS3  PHF23  C1orf55  LOC389137  SFN  LIF  ISG20L1  KTI12  PPP1R1B  SOCS3  LOC727901  IFNGR2  MPV17L2  CHD1  RANBP6  LOC650369  PMEPA1  CX3CL1  PIM2  TGM2  HIF1A  ACTBL2  LOC728115  SERTAD1  FAM84B  ARID4B  GCNT3  CDH5  PFKFB3  MAFF  PNO1  MGC4677  GRPEL1  ITPRIP  CDC42EP4  C5orf30  DUSP4  PPTC7  FAM89A  RAB30  LYSMD2  CP110  NUFIP2  PDZD2  CD46  BAK1  NRIP1  FAM43A  PTPN12  IRF2BP2  LMCD1  TNFRSF21  RNY5  MGAT2  NOP56  TRAF4  ZBED5  MIR1974  MIR614  HMOX2  CTR9  NNMT  SLC30A1  LOC728969  PDE4B  STAT3  ZNF326  SLC25A25  LOC648343  MGC3032  SLC35C1  S100A7  C7orf43  WDR43  FGA  LNX2  VTRNA1-1  IFNGR1  LOC100129685  SLC1A5  POP1  C9orf62  RBM47  CD3EAP  LONRF1  LOC644852  ID4  SCYL3  CTGF  FBXO32  SOX13  ZNF483  FBXO2  GRIPAP1  PVRL4  UCP2  JARID2  TMEM137  PLEKHF1  TSC22D3  ANKRD20A1  DDIT4L  STARD13  LOC643031  HIST1H3D  NAB2  ZMYND8  GOLSYN  ANKRD20A1  ITGB5  WEE1  IRX5  HES1  C1orf106  OSR2  NUCKS1  LOC730024  TOB1  TRIL  SCYL3  KLF11  RPS29  CYTH2  C7orf54  BRWD1  LOC647488  LOC728499  KLF11  CCNG2  PLIN5  DUSP8  EDN2  PILRB  CBX4  DLX3  HOXC13  DLX1  CYR61  LFNG  CYP1B1  ZNF750  SMAD6 | NM_021643.1  NM_173475.1  NM_006113.3  NM_018022.1  NM_005238.2  NM_001005376.1  NM_052815.1  NM_006532.2  NM_004139.2  NM_003877.3  NM_005252.2  NM_172037.2  NM_001706.2  NM_025195.2  NM_014149.2  NM_144590.1  NM_021242.3  NM_023016.2  NM_001024809.2  XM_291277.4  NM_001080826.1  NM_145753.1  NM_006399.2  NM_183422.1  NM_003844.2  NM_198282.1  NM_006086.2  NM_002229.2  NM_033409.2  NM_002252.3  NM_021960.3  NM_012482.3  NM_181339.1  NM_002035.1  NM_004281.3  NM_002970.1  NM_144726.1  NM_033306.2  NM_005627.3  NM_004099.4  NM_012124.1  NM_000877.2  NM_004419.3  NM_004154.3  NM_005627.2  NM_006745.3  NR_003697.1  NM_182919.1  NM_000700.1  NM_031415.1  NM_000950.1  NM_015435.3  NM_145753.1  NM_022119.3  NM_000422.1  NM_033397.2  NM_030666.2  NM_173854.4  NM_001993.2  NM_014918.3  NM_153331.2  NM_032509.2  NM_004431.2  NM_176814.3  NM_001423.1  NM_030819.2  NM_022157.2  NM_002035.1  NM_018948.2  NM_032334.1  NM_015570.1  XR_038546.1  NM_182972.1  NM_020870.2  NM_001491.2  NM_024873.2  NM_001634.4  NM_004591.1  XM_001722644.1  NM_031307.2  NM_020892.1  NM_021238.2  NM_020368.1  NM_006868.2  NM_005841.1  NM_000418.2  NM_024616.1  NR_001283.1  NM_000201.1  NM_013248.2  NM_138395.2  NM_015892.2  NM_024881.3  XR_038835.1  XM_376787.3  XM_941654.1  NM_002252.3  NM_024297.1  NM_152608.2  XM_371655.2  NM_006142.3  NM_002309.2  NM_022767.2  NM_138417.2  NM_181505.1  NM_003955.3  XR_042470.1  NM_005534.2  NM_032683.1  NM_001270.2  NM_012416.2  NM_021238.1  NM_199169.1  NM_002996.3  NM_006875.2  NM_004613.2  NM_181054.1  NM_001017992.2  XR_038319.1  NM_013376.1  NM_174911.3  NM_016374.5  NM_004751.1  NM_001795.2  NM_004566.2  NM_012323.2  NM_020143.2  NM_052871.3  NM_025196.2  NM_033397.2  NM_012121.4  NM_033211.2  NM_001394.5  NM_139283.1  XM_939093.1  NM_014488.3  NM_153374.1  NM_014711.3  NM_020772.1  NM_178140.1  NM_172358.1  NM_001188.2  NM_003489.2  NM_153690.4  NM_002835.2  NM_182972.2  NM_014583.2  NM_014452.3  NR_001571.2  NM_002408.3  XM_936090.1  NM_004295.2  NM_021211.2  NR_031738.1  NR_030345.1  NM_002134.2  NM_014633.2  NM_006169.2  NM_021194.2  XM_001132928.1  NM_002600.3  NM_139276.2  NM_182975.1  NM_052901.2  XM_937384.1  Hs.568944  NM_018389.3  NM_002963.2  NM_018275.3  XM_944889.1  NM_021871.2  NM_153371.2  NR_026703.1  NM_000416.1  XM_001723814.1  NM_005628.1  NM_015029.1  XM_926593.1  NM_001098634.1  NM_012099.1  NM_152271.2  XM_934218.1  NM_001546.2  NM_181093.1  NM_001901.2  NM_058229.2  NM_005686.2  NM_001007169.1  NM_183412.2  NM_207672.1  NM_030916.1  NM_003355.2  NM_004973.2  NM_032886.1  NM_024310.2  NM_004089.3  NM_001012419.1  NM_145244.2  NM_178008.1  XM_926402.1  NM_003530.3  NM_005967.2  NM_183048.1  NM_017786.2  NM_032250.1  NM_002213.3  NM_003390.2  NM_005853.4  NM_005524.2  NM_018265.1  NM_053001.1  NM_022731.2  XM_928770.1  NM_005749.2  NM_014817.2  NM_020423.4  NM_003597.4  NM_001030001.1  NM_017457.4  NM_014411.2  NM_033656.2  XM_942862.1  XM_928299.1  XM_938887.1  NM_004354.1  NM_001013706.2  NM_004420.1  NM_001956.2  NM_175047.2  NM_003655.2  NM_005220.2  NM_017410.2  NM_178120.3  NM_001554.3  NM_002304.1  NM_000104.2  NM_024702.1  NM_005585.2 | 1.70  1.69  1.69  1.68  1.67  1.67  1.67  1.65  1.65  1.64  1.64  1.64  1.64  1.63  1.63  1.63  1.61  1.61  1.61  1.60  1.59  1.58  1.57  1.57  1.55  1.54  1.54  1.54  1.53  1.53  1.53  1.52  1.52  1.51  1.51  1.50  1.50  1.50  1.50  1.49  1.49  1.48  1.48  1.47  1.47  1.47  1.47  1.46  1.46  1.45  1.45  1.45  1.45  1.45  1.45  1.45  1.44  1.44  1.44  1.44  1.44  1.44  1.43  1.43  1.43  1.43  1.43  1.43  1.43  1.43  1.42  1.42  1.42  1.42  1.42  1.41  1.41  1.41  1.41  1.41  1.41  1.41  1.40  1.40  1.40  1.40  1.40  1.40  1.40  1.40  1.40  1.39  1.39  1.39  1.39  1.39  1.39  1.39  1.38  1.38  1.38  1.38  1.38  1.38  1.38  1.38  1.37  1.37  1.37  1.37  1.37  1.37  1.37  1.36  1.36  1.36  1.36  1.36  1.36  1.36  1.36  1.36  1.36  1.36  1.35  1.35  1.35  1.35  1.35  1.35  1.34  1.34  1.34  1.34  1.34  1.33  1.33  1.33  1.33  1.33  1.33  1.32  1.32  1.32  1.32  1.32  1.32  1.32  1.32  1.32  1.32  1.32  1.32  1.32  1.32  1.31  1.31  1.31  1.31  1.31  1.31  1.31  1.31  1.31  1.31  1.31  1.31  1.31  1.31  1.31  1.31  1.31  1.31  1.31  1.31  1.31  1.30  1.30  1.30  1.30  1.30  0.77  0.77  0.77  0.77  0.76  0.76  0.76  0.76  0.76  0.76  0.76  0.75  0.75  0.75  0.75  0.75  0.75  0.75  0.75  0.75  0.75  0.75  0.75  0.74  0.74  0.73  0.73  0.73  0.73  0.73  0.72  0.72  0.72  0.71  0.71  0.71  0.71  0.71  0.70  0.70  0.70  0.70  0.69  0.69  0.68  0.67  0.67  0.66  0.66  0.65  0.65  0.64  0.63  0.61  0.61  0.60  0.59 | 1.95E-03  5.76E-03  6.51E-04  1.80E-03  6.20E-03  1.77E-04  1.93E-02  7.59E-04  1.31E-02  1.41E-03  5.08E-03  2.14E-03  5.50E-03  1.08E-03  3.08E-05  2.78E-03  1.90E-02  7.08E-03  2.36E-03  8.28E-03  1.58E-04  3.37E-03  1.59E-03  4.42E-03  5.12E-02  1.16E-04  4.26E-05  1.18E-02  1.49E-03  3.47E-04  2.41E-02  1.93E-04  4.21E-02  3.17E-03  9.38E-03  1.16E-03  2.78E-02  2.84E-03  9.49E-04  3.52E-03  3.00E-02  2.01E-02  4.77E-02  1.44E-02  1.05E-02  1.05E-02  1.11E-03  1.29E-02  2.55E-03  1.02E-04  3.67E-05  3.06E-03  4.84E-03  7.63E-05  1.76E-02  6.29E-04  3.28E-04  3.11E-03  1.79E-02  1.72E-03  2.22E-02  2.06E-02  2.42E-02  2.08E-02  6.30E-03  1.10E-02  1.02E-03  1.52E-03  2.17E-02  1.32E-02  7.35E-04  1.18E-02  3.57E-03  5.31E-03  3.54E-03  3.24E-02  2.32E-02  3.63E-03  5.72E-02  1.12E-02  2.86E-03  8.00E-03  2.53E-02  2.43E-04  1.18E-02  1.65E-03  2.21E-02  2.52E-02  3.67E-02  4.30E-04  1.77E-02  2.09E-04  1.47E-02  4.02E-02  5.46E-02  1.06E-02  3.18E-02  3.42E-02  3.94E-04  5.26E-02  4.54E-02  1.83E-02  5.50E-03  8.98E-04  1.06E-03  3.62E-03  1.08E-04  2.09E-03  3.43E-04  3.24E-02  3.23E-02  7.41E-03  5.46E-04  4.18E-03  3.95E-04  1.13E-02  7.39E-02  2.00E-02  5.99E-02  7.00E-04  6.82E-02  1.30E-01  2.07E-02  2.48E-02  2.02E-04  1.04E-03  3.55E-02  4.39E-03  2.47E-02  1.25E-02  3.60E-02  1.88E-03  3.01E-02  2.59E-02  3.45E-03  1.70E-02  3.72E-03  3.17E-02  1.33E-02  6.69E-03  1.19E-01  4.57E-02  9.10E-03  1.11E-03  4.46E-02  1.64E-02  5.76E-02  2.87E-02  1.61E-01  3.17E-03  2.38E-02  3.20E-03  4.79E-02  4.26E-01  1.12E-02  3.34E-02  5.87E-03  1.30E-02  3.56E-02  1.07E-02  1.49E-02  5.67E-02  1.74E-01  2.49E-02  1.62E-01  3.95E-03  1.57E-03  1.55E-01  1.19E-04  2.65E-02  6.33E-03  1.40E-01  1.24E-01  5.35E-03  6.67E-02  1.29E-01  3.42E-03  3.19E-02  1.08E-01  1.87E-02  2.45E-02  1.48E-01  3.03E-04  3.49E-04  2.40E-02  1.72E-02  3.90E-03  2.47E-01  4.72E-01  2.87E-01  4.09E-02  3.77E-02  2.69E-03  7.29E-02  1.42E-01  1.54E-02  4.85E-02  1.13E-02  1.49E-03  2.38E-01  2.31E-02  8.07E-03  2.36E-02  5.58E-03  8.95E-02  1.28E-02  1.85E-02  1.86E-02  5.44E-02  2.92E-03  8.39E-03  1.06E-01  2.25E-02  2.78E-02  5.50E-03  8.34E-04  3.32E-02  6.63E-02  2.69E-02  1.83E-01  2.48E-03  3.22E-01  1.75E-02  4.33E-03  6.77E-03  1.25E-01  6.05E-03  4.62E-03  6.66E-02  6.90E-04  2.18E-03  1.64E-03  6.50E-03  8.52E-03  2.08E-03  6.98E-05  1.33E-02  1.99E-03 | | MAPK regulation  Cell cycle control, cell growth  Angiogenesis  Transcription  Receptor for urokinase plasminogen activator  Anti-apoptosis  Transcription  Immune response  Cytokine signal transduction  Signal transduction  Retinol dehydrogenase  Transcription  MAPK regulation  Lipogenesis  Transcription  Tyrosine kinase activity  Tyrosine kinase activity  Phospholipid binding  Transcription  Transcription  TNF receptor  Transcription  Cytoskeleton  Transcription  Potassium channel  Apoptosis  Transcription  Apoptosis  Anti-apoptosis  Metabolism  Apoptosis  Serine/Threonine kinase activity  Ion channels  Chaperone activity  Signal transduction  MAPK pathway inhibition  G-protein coupled receptor activity  Serine/Threonine kinase activity  Oxidase activity  Signal transduction  Phospholipid binding  Calcium ion binding  Transcription  Phospholipid binding  Serpin protease  Cytoskeleton  Protein kinase inhibitor activity  Proteinase inhibition  Cation transmembrane transporter activity  Phospholipid binding, protease binding  Metabolism  Potassium channel  Ephrin receptor activity  Transcription  Matrix assembly  GTPase activity  Reductase activity  Rho GTPase activator activity  Ribosome biogenesis  Transcription  Signal transduction  Polyubiquitin binding  Decarboxylase activity  Chemokine activity  Pseudogene  Pseudouridine synthase activity  Ligase activity  Transcription  Gene silencing  GDP and GTP binding  Antagonist of FGF pathways  IL-4 receptor activity  Pseudogene  Integrin binding  Rna GTPase binding  Methionine-tRNA ligase activity  Sulfotransferase activity  Putative transporter  Pseudogene  Pseudogene  Phosphatase binding and actin binding  Potassium channel activity  Pseudogene  Protein kinase binding  Growth factor activity  Exonuclease activity  Protein kinase inhibitor activity  Protein kinase inhibitor activity  IFN-gamma receptor activity  ATP-dependent DNA helicase activity  Nuclear transport receptor  WW domain binding  Chemokine activity  Protein serine/threonine kinase activity  Transglutaminase activity  Transcription  Actin, cytoskeleton  Transcription  Transcription  Acetylglucosaminyltransferase activity  Ion channel binding, cell adhesion  Metabolism  Transcription  RNA binding  Chaperone binding  Protein kinase inhibitor activity  GTP-Rho binding, cytoskeleton  Protein trafficking  Protein tyrosine/threonine phosphatase activity  Phosphoprotein phosphatase activity  GTPase activity  Cytokinesis regulation  RNA binding  Complement binding  Protein dimerization activity, apoptosis  Transcription  Protein tyrosine phosphatase activity  Transcription  Transcription  Apoptosis  Acetylgluocosaminyltransferase activity  RNA binding  Protein kinase binding, apoptosis  MicroRNA  MicroRNA  Heme oxygenase activity  SH2 domain binding  Nicotinamide N-methyltransferase activity  Zinc ion transmembrane transporter activity  Ion channel binding  Transcription  RNA polymerase II core binding  Calcium ion binding  Fucose transporter  RAGE receptor binding  Protein binding, bridging, receptor binding  PZD domain binding  Cytokine binding, IFN-g receptor activity  Neutral amino acid transporter activity  Ribonuclease MPR activity  RNA binding  DNA-directed RNA polymerase activity  ATP-dependent peptidase activity  Transcription  Kinase activity, cell adhesion/migration  Integrin binding, cell proliferation  Transcription  Transcription  Ubiquitin-protein ligase, glycoprotein binding  Guanine nucleotide exchange factor  Cell adhesion  Mitochondrial anion carrier protein  Chromatin binding, histone demethylase activity  RNA binding, protein binding, bridging  Phosphatidylinositol-3-phosphate binding  Transcription  Regulation of mTOR signaling pathway  GTPase activator activity  Core component of nucleosome  Transcription  Transcription  Kinesin binding, syntaxin-1 binding  Integrin binding, receptor activity  Protein serine/threonine kinase activity  Transcription  Transcription  Transcription  Transcription  Lipopolysaccharide binding  Kinase activity, cell adhesion/migration  Transcription  Ribosome constituent  Phospholipid binding  Transcription  Pseudogene  Transcription  Cell cycle control  Inhibition of lipolytic degradation  MAP kinase phosphatase activity  Hormone activity  Immune response  Transcription  Transcription  Transcription  Transcription  IGF binding, cell proliferation, chemotaxis, angiogenesis, cell adhesion  Glycosyltransferase activity  Electron carrier activity, metabolism  Transcription  Transcription | |  |
| CCNG2 | NM_004354.1 | 0.58 | 2.05E-04 | | Cell cycle regulation | |  |
| TXNIP | NM_006472.1 | 0.57 | 7.37E-03 | | Transcription | |  |
| EDN1 | NM_001955.2 | 0.55 | 9.08E-05 | | Vasoconstriction | |  |
| HERPUD1 | NM_001010990.1 | 0.53 | 1.50E-04 | | Ubiquitination | |  |
| CITED2 | NM_006079.3 | 0.52 | 5.18E-05 | | Transcription | |  |
| FBXO32 | NM_148177.1 | 0.49 | 1.46E-03 | | Ubiquitination | |  |
| FAM46B | NM_052943.2 | 0.42 | 4.07E-04 | |  | |  |
| LOC643009 | XM_932195.1 | 0.42 | 3.61E-01 | |  | |  |
| ID2 | NM_002166.4 | 0.33 | 4.67E-04 | | Transcription | |  |
| CYP1A1 | NM_000499.2 | 0.32 | 8.55E-04 | | Metabolism | |  |
| ID1 | NM_181353.1 | 0.29 | 3.33E-05 | | Transcription | |  |

Only genes with more than 1.3-fold changes are shown
