# Supplementary material for: Adipose cells promote resistance of breast cancer cells to trastuzumab-mediated antibody-dependent cellular cytotoxicity
Source: Breast Cancer Res. 2015 Apr 24;17(1):57. doi: 10.1186/s13058-015-0569-0 (PMC4482271; doi:10.1186/s13058-015-0569-0)
Supplement: Supplementary file 10 — Downregulation of GDF15, MYC and SERPINA3 by siRNA in ADCC assays. BT-474 cells were transfected with 10 nM scrambled siRNA or siRNA of indicated target genes for 48 hours. At 48 hours posttransfection, gene expression levels of target genes were analyzed by RT-qPCR (A) and BT-474 cells were used for ADCC assays (B) in the presence of the control medium or #hMADS-CM. The results shown are means ± SD of at least three independent experiments. [file 13058_2015_569_MOESM10_ESM.docx]

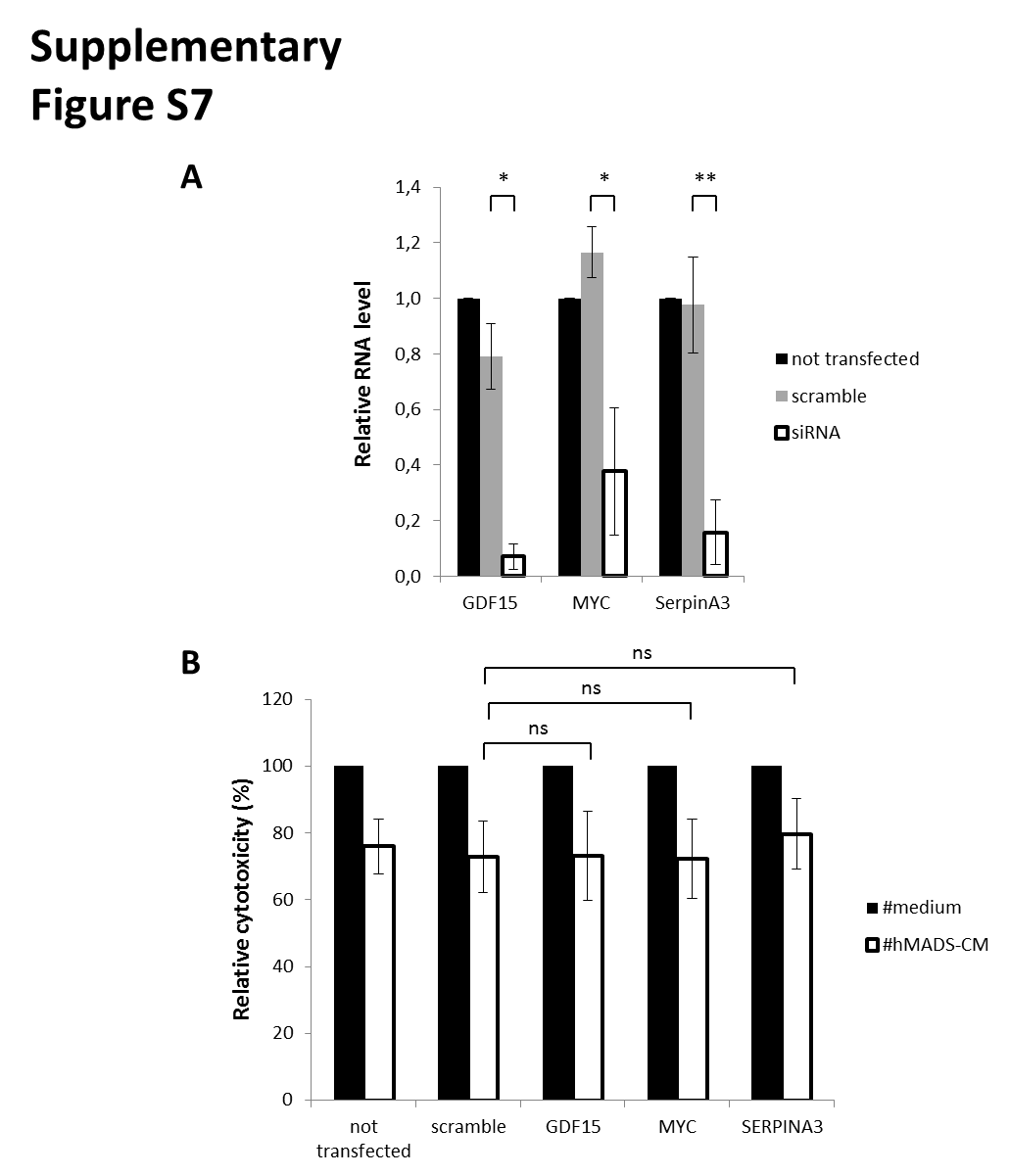


**Fig. S7. Down-regulation of GDF15, MYC and SERPINA3 by siRNA in ADCC assays**. BT474 cells were transfected with 10 nM scrambled siRNA or siRNA of indicated target genes for 48 h. At 48 h post-transfection, gene expression levels of target genes were analyzed by RT-qPCR (**A**) and BT474 cells were used for ADCC assays (**B**) in the presence of the control medium or #hMADS-CM. Results are means ± SD of at least 3 independent experiments. *p<0.05; **p<0.01; ns: not significant.
