# Supplementary material for: Adipose cells promote resistance of breast cancer cells to trastuzumab-mediated antibody-dependent cellular cytotoxicity
Source: Breast Cancer Res. 2015 Apr 24;17(1):57. doi: 10.1186/s13058-015-0569-0 (PMC4482271; doi:10.1186/s13058-015-0569-0)
Supplement: Supplementary file 11 — Protection of BT-474 cells by #hMADS-CM from T-DM1. BT-474 cells were exposed to the indicated concentrations of T-DM1 in the presence of the control medium or #hMADS-CM for 72 hours. Cell proliferation was determined by MTT assay. The results shown are representative of three independent experiments. [file 13058_2015_569_MOESM11_ESM.docx]

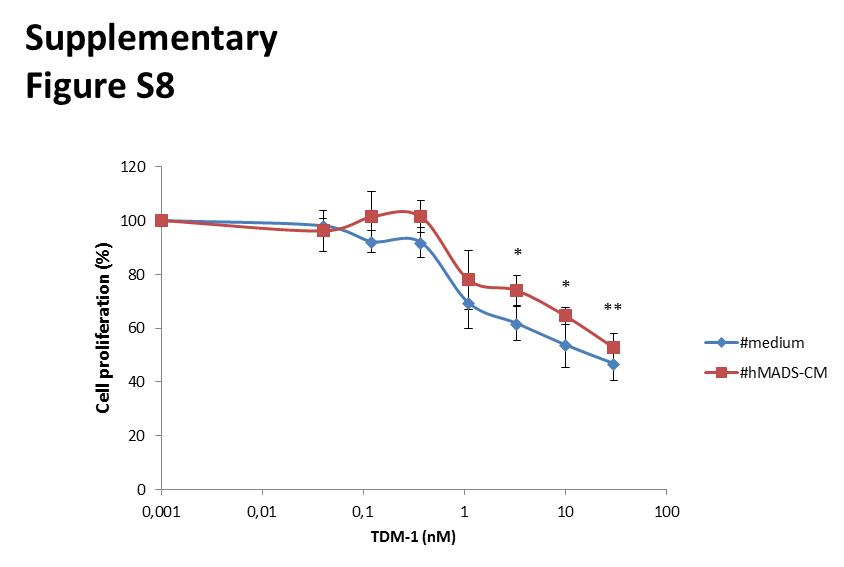


**Fig. S8. Protection of BT474 cells by #hMADS-CM from TDM-1**. BT474 cells were exposed to different concentrations of TDM-1 in the presence of the control medium or #hMADS-CM for 72 h. Cell proliferation was determined by MTT assay. Results are means ± SD of 4 independent experiments. *p<0.05; **p<0.01.
