# Supplementary material for: Adipose cells promote resistance of breast cancer cells to trastuzumab-mediated antibody-dependent cellular cytotoxicity
Source: Breast Cancer Res. 2015 Apr 24;17(1):57. doi: 10.1186/s13058-015-0569-0 (PMC4482271; doi:10.1186/s13058-015-0569-0)
Supplement: Supplementary file 12 — List of adipocyte-derived factors tested in ADCC assays. [file 13058_2015_569_MOESM12_ESM.docx]

**Supplementary Table 3. List of adipocyte-derived factors tested in ADCC assays**

| **Types** | **Factors tested** | **Approaches used** | **Providers** | **Concentrations** |
| --- | --- | --- | --- | --- |
| Hormone | Estrogen | Tamoxifen* | Sigma | 10 µM |
|  |  | Activated charcoal-treatment of CM* | Sigma | 10 mg/mL |
| Growth factor | IGF-1 | Recombinant protein | Tercica | 100-1000 pg/mL |
| Adipokine | Leptin | Recombinant protein | Sigma,  R&D Systems | 10-500 ng/mL |
|  | Adiponectin | Recombinant protein | Sigma,  R&D Systems | 10-100 µg/mL |
|  | IL-6 | Recombinant protein | Sigma | 10-100 ng/mL |
|  | TNF-alpha | Recombinant protein | Cetus Corp. | 10 ng/mL |
|  | TGF-beta | Recombinant protein | Sigma | 0.1-10 ng/mL |
|  | IL-1beta | Recombinant protein | Invivogen | 100-1000 ng/mL |
|  | IL-7 | Recombinant protein | Sigma | 50-500 ng/mL |
|  | GDF15 | Recombinant protein | R&D Systems | 0.1-1000 ng/mL |
| Serpin | SerpinA1/Alpha-antitrypsin | Purified protein | Sigma | 2-2000 µg/mL |
|  | SerpinA3 | Purified protein | Prospec | 0.1-250 µg/mL |
|  | SerpinA12/Vaspin | Recombinant protein | Preprotech | 100-1000 pg/mL |
|  | SerpinE1/PAI-1 | Recombinant protein | Millipore | 1-1000 ng/mL |
| Nucleoside | Adenosine | Synthetic molecule | Sigma | 10 µM |
|  |  | Adenosine analog (NECA) | Sigma | 10-50 µM |
|  | AMP, ADP | Synthetic molecule | Sigma | 1 mM |
|  | ATP | Synthetic molecule | Sigma | 1-1000 µM |
|  |  | Apyrase (ATPase)* | Sigma | 0.25 unit/mL |
| Lipid | LPA | Synthetic molecule | Avanti Polar Lipids | 10 µM |
|  | S1P | Synthetic molecule | Avanti Polar Lipids | 1 µM |
| Enzyme | CD73 | Inhibitor (APCP)* | Sigma | 100 µM |
|  | Autotaxin | Recombinant protein | Cayman Chemical | 50-300 ng/mL |

*added in the presence of #hMADS-CM to test the potential reversion of the inhibition of ADCC induced by #hMADS-CM
